# Stemness of the Organ of Corti Relates to the Epigenetic Status of Sox2 Enhancers

**DOI:** 10.1371/journal.pone.0036066

**Published:** 2012-05-03

**Authors:** Jörg Waldhaus, Jelka Cimerman, Henning Gohlke, Mathias Ehrich, Marcus Müller, Hubert Löwenheim

**Affiliations:** 1 Department of Otorhinolaryngology, Head and Neck Surgery, Hearing Research Center Tübingen, University of Tübingen Medical Center, Tübingen, Germany; 2 SEQUENOM GmbH, Hamburg, Germany; 3 SEQUENOM Inc., San Diego, California, United States of America; National Cancer Institute, United States of America

## Abstract

In the adult mammalian auditory epithelium, the organ of Corti, loss of sensory hair cells results in permanent hearing loss. The underlying cause for the lack of regenerative response is the depletion of otic progenitors in the cell pool of the sensory epithelium. Here, we show that an increase in the sequence-specific methylation of the otic Sox2 enhancers NOP1 and NOP2 is correlated with a reduced self-renewal potential *in vivo* and *in vitro*; additionally, the degree of methylation of NOP1 and NOP2 is correlated with the dedifferentiation potential of postmitotic supporting cells into otic stem cells. Thus, the stemness the organ of Corti is related to the epigenetic status of the otic Sox2 enhancers. These observations validate the continued exploration of treatment strategies for dedifferentiating or reprogramming of differentiated supporting cells into progenitors to regenerate the damaged organ of Corti.

## Introduction

In the functionally mature mammalian organ of Corti (OC), hair cell regeneration does not occur endogenously as it does in the hair cell epithelia of other vertebrates by evolutionarily conserved mechanisms such as morphallaxis or epimorphosis [1]. However, the functionally immature postnatal OC harbors a latent regenerative potential. This intrinsic regenerative potential is indicated by the presence of multipotent stem cells that, when isolated, can self-renew and differentiate into supporting and hair cell lineages, as demonstrated by otic sphere formation assays [2]–[6]. These stem cell-like properties have been ascribed to the supporting cell population of the postnatal OC. This conclusion is supported by the observation that when postnatal supporting cells are purified by fluorescence-activated cell sorting using approaches such as p27^Kip1^-GFP transgenic mice [7], side population analysis [8], selective surface markers on supporting cells [6] or Lgr5-GFP transgenic mice [9], they acquire stem cell-like properties similar to progenitor cells in the early embryonic OC. The present report focuses on otic spheres and stem cells derived from the postnatal OC, herein referred to as organ of Corti derived stem cells (OCSCs). A loss of OCSC isolation capacity and regenerative potential of isolated supporting cells is seen after the second postnatal week [4], [7]. It is presumed that this loss is caused by a depletion of endogenous stem/progenitor cells or by a loss of intrinsic regenerative properties from the pool of supporting cells in the sensory epithelium. Therefore, it is of particular interest to understand how the postnatal presence of isolatable stem cells and the loss of this capacity in the mature organ are controlled at the molecular level.

The HMG-box transcription factor Sox2 functions with Oct4 and Nanog to maintain the pluripotency of embryonic stem cells (ESCs) [10]. Remarkably, the forced expression of Sox2 in combination with Oct4, cMyc and Klf4 induces the formation of pluripotent stem cells (iPSCs) from differentiated somatic cells [11]. In ESCs, Sox2 functions as a molecular rheostat because tightly regulated Sox2 levels control the expression of critical subsets of genes, thereby stimulating the opposing phenomena of either self-renewal or differentiation [12]. A similar dual function is seen in neural stem cells (NSCs); here, Sox2 is required to maintain “stemness” [13], but it also controls the seemingly opposed differentiation of distinct cell types in the eye [14] and brain [15], which indicates that Sox2 has dose- and context-dependent functions [16], [17]. In the OC, Sox2 also appears to serve a dual role in establishing progenitors in the prosensory domain [18] and the subsequent differentiation of supporting cells [19]. In the vestibular epithelium, Sox2 has also been described to function in both sphere formation and differentiation of inner ear stem cells derived from the utricle [20].

Numerous studies suggest that the complex regulation of Sox2 in ESCs, NSCs and potentially OCSCs is influenced by the activity of Sox2 enhancer elements [21]–[26]. The Sox2 enhancers SRR1 and SRR2 are known to exert their activities in ESCs [17], [24] and NSCs [22]. Notably, a reporter-based assay revealed that two enhancer elements, NOP1 and NOP2, (Figure S1A, B) are uniquely activated in nasal and otic placodes during chicken development [25]. The sequences of these functionally identified otic Sox2 enhancers correspond to extragenic sequence blocks, that are conserved in chickens (Chicken Sox2: GenBank: AB092842.1), mice (Mouse chromosome 3 including Sox2: GenBank: AL606746.17) and humans [25] (Human chromosome 3 including Sox2: GenBank: AC117415.7) (Table S1, Figure S1A, B). Two mouse mutants, *Ysb* and *Lcc* (Figure S2A, B), represent unique alleles of Sox2 in which complex chromosomal rearrangements have resulted in the compromised function of specific enhancers that direct Sox2 expression in the inner ear [18]. Findings in these mutants indicate a critical role for the tissue-specific Sox2 enhancers in the establishment and maintenance of otic progenitors in the sensory primordium during development [18]. In fact NOP1 enhancer activity has been directly demonstrated in the otic placode of a primary transgenic mouse model at embryonic day (E) 9.5 (Kondoh H, pers. communication). In summary, these findings suggest a putative role for the NOP1 and NOP2 enhancers in regulating the stemness of the OC.

In this study, the molecular signature of OCSCs, NSCs and ESCs revealed similarities between the OCSCs and the NSCs, with the exception of the SRR1/SRR2 and NOP1/NOP2 enhancer status. During OC development, the Sox2 promoter remained demethylated, whereas the otic enhancers NOP1 and NOP2 were subject to progressive methylation. The OCSCs maintained an otic Sox2 enhancer methylation pattern that resembled differentiating postnatal supporting cells. A pronounced, sequence-specific methylation of NOP1 and NOP2 enhancers was observed in relation to differentiation *in vivo* and *in vitro*. In addition, NOP1 and NOP2 enhancer methylation in OCSCs was induced by EGF stimulation and predominately resulted in a previously characterized non-self-renewing hollow otic sphere phenotype [27]. Overall the epigenetic status of the otic Sox2 enhancers NOP1 and NOP2 reflected the stemness of the embryonic and early postnatal OC and OCSCs.

## Results

### OCSCs resemble a multipotent NSC state rather than a pluripotent ESC state

To define the molecular signature of OCSCs, the DNA methylation pattern within the promoter regions of the three key pluripotency genes (Sox2, Oct4 and Nanog) was analyzed applying a quantitative methylation approach using bisulfite conversion and quantitative methylation analysis (EpiTyper).

**Figure 1:**
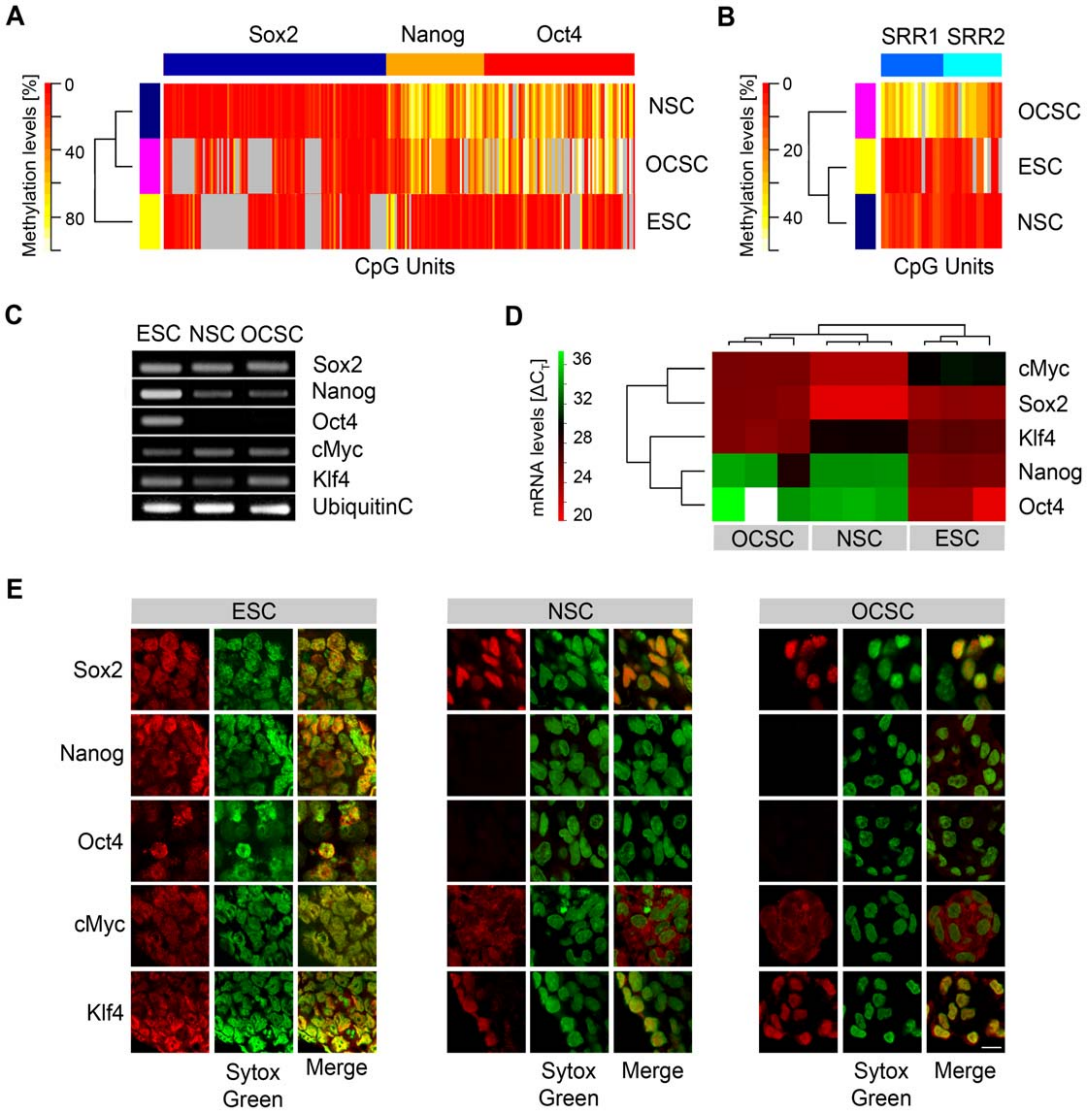
Comparative analysis of the pluripotency factors Sox2, Nanog, Oct4, cMyc and Klf4 in ESCs, NSCs and OCSCs. (**A**) Bisulfite methylation profiles for Sox2, Nanog and Oct4 promoters in ESCs, NSCs and OCSCs. (**B**) Bisulfite methylation profiles for the Sox2 enhancers SRR1 and SRR2 in ESCs, NSCs and OCSCs. DNA methylation values are depicted on a pseudo-color scale as indicated (methylation increases from red [non-methylated] to yellow [methylated]); missing values are shown in grey. (**C**) RT-PCR analysis of pluripotency marker expression in ESCs, NSCs and OCSCs. Ubiquitin C was used as the loading control. (**D**) qPCR analysis for the same five factors as in (**C**) in ESCs, NSCs and OCSCs. ΔC_T_ values were normalized to HPRT1/TbP, compared using a Pearson's Correlation and displayed in a heat map. Red indicates up-regulation with a ΔC_T_ value below the mean level the analyzed dataset, and green indicates down-regulation with a ΔC_T_ value above the mean level (i.e., see also Figure S3, Table S2A). (**E**) Immunocytochemistry of ESCs, NSCs and OCSCs. Identical settings were used for image acquisition (Scale Bar: 10 µm).

Previous studies have shown that OCSCs isolated from the OC can self-renew and differentiate into supporting and hair cell-like cells, which is consistent with a multipotent stem cell state [4], [6], [27], [28]. For a comparative classification, we also analyzed multipotent NSCs from the postnatal forebrain and pluripotent ESCs from the same genetic background. The Sox2 promoter was highly demethylated in all three stem cell populations (ESCs 7%; NSCs 6%; OCSCs 10%) (Figure 1A). The Nanog and Oct4 promoters were also demethylated in ESCs (12% and 11%, respectively), whereas the same promoters were methylated in NSCs (54% and 50%, respectively) and OCSCs (35% and 58%, respectively) (Figure 1A). Overall cluster analysis of all three promoters demonstrated that OCSCs and NSCs featured a similar epigenetic pattern, that differed from that observed in ESCs (Figure 1A). However, differences in the tissue-specific epigenetic landscape of OCSCs and ESCs/NSCs were evident in our analysis of the ESC- and NSC-specific Sox2 enhancers SRR1 and SRR2 [22], [24]. A cluster analysis of SRR1 and SRR2 confirmed a similar demethylated pattern for ESCs (5% and 8%, respectively) and NSCs (6% and 4%, respectively), whereas the same enhancers were methylated in OCSCs (45% and 37%, respectively) (Figure 1B). Transcripts for the pluripotency-related genes Sox2, Nanog, Oct4, Klf4 and cMyc were determined in ESC, NSC and OCSC preparations using Reverse Transcription Polymerase Chain Reaction (RT-PCR). All pluripotency-related mRNAs were expressed in all three stem cell populations, except Oct4, which was not detected in OCSCs and NSCs (Figure 1C).

OCSCs, NSCs and ESCs were further compared at the transcriptional level using quantitative PCR (qPCR) (Figure 1D, Table S2A). The data were normalized to positive-control ESCs that expressed all five ESC marker genes (i.e., Sox2, Nanog, Oct4, cMyc and Klf4). We determined that Nanog and Oct4 were silenced in OCSCs (0.018-fold and 0.004-fold, respectively) and NSCs (0.004-fold and 0.001-fold, respectively), whereas Sox2, cMyc and Klf4 were expressed in both stem cell populations (Table S2A). Overall, a cluster analysis of all pluripotency-related genes demonstrated a transcriptional pattern that was similar for OCSCs and NSCs but differed from that of ESCs (Figure 1D).

At the protein level, immunocytochemical labeling of Sox2, Nanog, Oct4, cMyc and Klf4 demonstrated nuclear expression of Sox2 and Klf4 in all three stem cell populations; however, Nanog, Oct4 and cMyc expression was confirmed only in ESCs (Figure 1E).

### Otic Sox2 enhancers are subject of epigenetic regulation during OC development

The comparative analyses of the three different stem cell types raised the question to what extent the developmental decrease in OCSC isolation capacity [4] and the related decrease in stemness [7] relates to Sox2 and its epigenetic transcriptional regulation.

**Figure 2:**
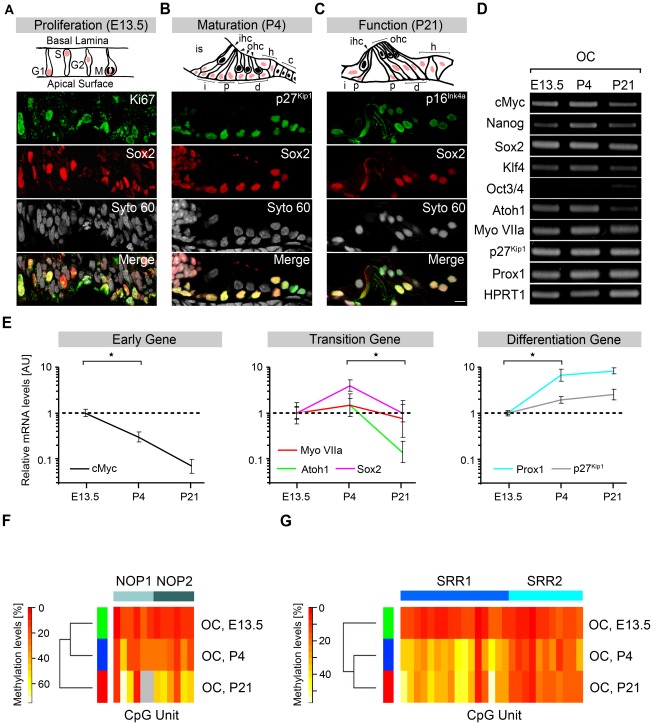
Epigenetic, transcriptional and translational characterization of Sox2 expression during OC development. (**A**–**C**) OC during development. (**A**) Upper panel: Schematic of the sensory domain, which contains the proximal cochlea duct, showing interkinetic nuclear migration at E13.5. Sox2 expression is indicated by red nuclei. Remaining panels: marker expression at E13.5. All proliferating Ki-67-positive cells are co-labeled for Sox2. (**B**) Upper panel: schematic of the different cell types found in the maturating OC at P4. Inner hair cell (ihc, arrowhead), three outer hair cells (ohc, arrowheads) and different supporting cells: inner sulcus cells (is); interphalangeal cells (i); pillar cells (p); Deiters' cells (d); Hensen's cells (h); and Claudius cells (c). Remaining panels: marker expression at P4. The quiescence of Sox2-positive supporting cells is indicated by co-labeling with p27Kip1. (**C**) Upper panel: schematic of the different cell types found in the functional OC at P21. Remaining panels: marker expression at P21. Senescence of Sox2-positive cells is indicated by p16Ink4a expression. (**D**) RT-PCR of pluripotency marker, hair cell marker and supporting cell marker expression in the OC (E13.5, P4, P21). HPRT1 was used as the loading control. (**E**) qPCR analysis of six developmentally regulated genes (cMyc, Sox2, Atoh1, Myosin VIIa, p27Kip1 and Prox1) during OC development (E13.5, P4 and P21). The relative expression levels of P4 and P21 were compared with those at E13.5. The transcript levels were normalized to HPRT1/Ubiquitin C levels. Averages of the three independent experiments with SDs are shown (*p<0.05) (i.e., see also Table S2B). Depending on the temporal expression pattern, genes were assigned to early, transition or differentiation groups (i.e., see also Figure S3). (**F**,**G**) Bisulfite methylation of the Sox2 enhancers (f) (NOP1/2) and (g) (SRR1/2) during OC development (E13.5, P4, P21) (i.e., see also Figure S4). (Scale Bars: A,B,C, 10 µm).

Hence, the developmental pattern of Sox2 protein expression was analyzed to identify Sox2 in the following three types of cells: embryonic proliferating otic progenitors, postnatal maturing supporting cells and fully differentiated supporting cells at the functionally mature stage. At E13.5, nuclear Sox2 expression was co-localized with the proliferation marker Ki-67, indicating the presence of Sox2-positive proliferating progenitors in the prosensory domain of the proximal cochlear duct (Figure 2A). At postnatal day 4 (P4), in the maturing OC, nuclear Sox2 expression was co-localized with the cell cycle inhibitor p27^Kip1^ in postmitotic supporting cells (inner phalangeal cells, pillar cells, Deiters' cells, Hensen's cells) (Figure 2B). This pattern of Sox2 expression and co-localization with p27^Kip1^ was maintained in the supporting cells of the functionally mature epithelium at P21, which is devoid of any stem cell isolation capacity [4] or regenerative potential in its supporting cells at P14 [7]. At postnatal day 4 weak expression of p16^Ink4a^ was detectable in a subset of Sox2-positive supporting cells (Figure S5B). At the functionally mature P21 stage, all of the different supporting cell types of the OC showed co-localization of Sox2 with the senescence marker p16^Ink4a^ (Figure 2C) indicating terminal differentiation and replicative senescence [29] of supporting cells at this time point. These findings demonstrate that Sox2 expression is maintained during three different states of the cell cycle in cochlear supporting cells at three different developmental time points.

To explore the function of Sox2 in stemness and differentiation, a comparative characterization of Sox2 expression during OC development was performed. Eight additional genes were assessed in parallel. The pluripotency-associated factors cMyc, Nanog, Klf4 and Oct4 were analyzed and considered indicative of stemness [30]. Hair cell differentiation was represented by the transcription factor Atoh1 [31], [32] and myosin VIIa [33]. Supporting cell differentiation was indicated by expression of the cell cycle regulator p27^Kip1^ [34] and Prox1 [35]. RT-PCR confirmed the transcription of all stemness- and differentiation-related genes, except Oct4, during OC development (Figure 2D).

To quantify differential gene expression, the relative abundance per single gene transcript was evaluated at the three developmental time points (i.e., E13.5, P4, P21) using qPCR. Based on the relative developmentally induced changes, transcripts were classified into three groups of developmentally regulated genes (early, transition, differentiation genes) as previously described in a different context [36] (see Figure S3, Table S2B and Supplemental Experimental Procedures S1). To facilitate the comparative analysis, data were normalized to the E13.5 progenitor stage. Each developmental stage was represented by a distinct expression pattern of the analyzed genes (Figure 2E). The proliferating primordium (E13.5) was characterized by a basal level of transition genes, such as Sox2 and Atoh1, a high level of the early gene cMyc and low levels of the differentiation genes Prox1 and p27^Kip1^. The postmitotic maturing OC (P4) showed a 3.9-fold (p<0.01) increase in the level of the transition gene Sox2 and maximum expression of the antagonistic factor Atoh1 (Figure 2E, Table S2B). In the functionally mature epithelium (P21), Sox2 was down-regulated (p<0.01), as compared to P4, back to the basal level observed at E13.5. At P21 the differentiation genes p27^Kip1^ and Prox1 reached maximum levels, while the early gene cMyc dropped to its lowest level (Figure 2E, Table S2B). Nanog and Oct4 were classified as low copy number or background genes (Figure S3D), and Klf4 was classified as a non-differentially regulated gene. Consequently, Nanog, Oct4 and Klf4 were not assigned to any of the investigated groups (Table S2B). These results indicate that Sox2 expression is developmentally regulated and carefully titrated. A 4-fold transcriptional up-regulation (Figure 2E) was correlated with the transition from proliferating progenitors at E13.5 to differentiating, quiescent supporting cells at P4 (Figure 2A, B). However, at P21 in the functionally mature organ Sox2 nuclear protein expression was maintained (Figure 2C), but a significant down-regulation occurred at the transcriptional level (Figure 2E).

To investigate the epigenetic mechanisms that might control Sox2 expression levels, the CpG methylation status of the Sox2 promoter was compared to that of the Nanog and Oct4 promoters during OC development. CpG islands within the Sox2 promoter remained demethylated at all three investigated time points (E13.5, 4%; P4, 7%; P21, 8%) (Figure S4A), which enabled constitutively active Sox2 transcription. Over the same time course, increased promoter silencing was seen for Nanog (E13.5, 15%; P4, 34%; P21, 46%) and Oct4 (E13.5, 28%; P4, 42%; P21, 57%) (Figure S4A). In contrast to the Sox2 promoter, the otic Sox2 enhancer elements NOP1 and NOP2 [25] showed a moderate increase in methylation during the early phase of OC development. This increase resulted in a clustering of E13.5 (NOP1, 12%; NOP2, 11%) with P4 (NOP1, 24%; NOP2, 24%), whereas progressive methylation was found for the mature P21 development stage (NOP1, 37%; NOP2, 39%) (Figure 2F). The further increase in NOP1 and NOP2 methylation from P4 to P21 correlated with the down-regulation of Sox2 mRNA to basal levels (Figure 2E). Interestingly, the SRR1 and SRR2 enhancers, which are supposedly not involved in otic development, follow a different time course of methylation during OC development (Figure 2G), resulting in a clustering of the P4 methylation pattern with the pattern at P21 (E13.5: SRR1, 10%; SRR2, 10%; P4: SRR1, 32%; SRR2, 17%; P21: SRR1, 36%; SRR2, 16%). In summary, the demethylated status of the Sox2 promoter enables for constitutive Sox2 expression in otic supporting cells, whereas modifications in the methylation of the otic enhancer elements might contribute to the fine titration of the Sox2 expression levels during development.

Promoter and regulatory elements in general are known to serve as integration sites of upstream signaling. Here, a sequence analysis of the NOP1 and NOP2 enhancer elements identified numerous DNA-binding motifs (Figure S1A, B). Because three potential SOX/LEF factor-binding sites [37] occur in the NOP1 and NOP2 sequences, it is possible that those enhancers are activated by Sox2 (Figure S1A, B). Additional binding sites in both enhancers include δEF1/SIP1 motifs [38] that overlap with E2 motifs, homeodomain protein binding sites and E-box motifs (Figure S1A, B); however, the significance of these predicted binding sites remains to be examined.

### Generation of multipotent otospheres does not affect the methylation status of Sox2 enhancers

Because Sox2 is thought to function in a dose-dependent manner, the methylation status of its enhancers may contribute to the regulation of Sox2 expression. A combination of dose- and context-dependent Sox2 functions may contribute to the developmental changes in the sphere isolation capacity of the OC. To test this hypothesis, we investigated whether the Sox2 enhancer is subject to epigenetic modification during the generation of OCSCs in an otic sphere assay.

**Figure 3:**
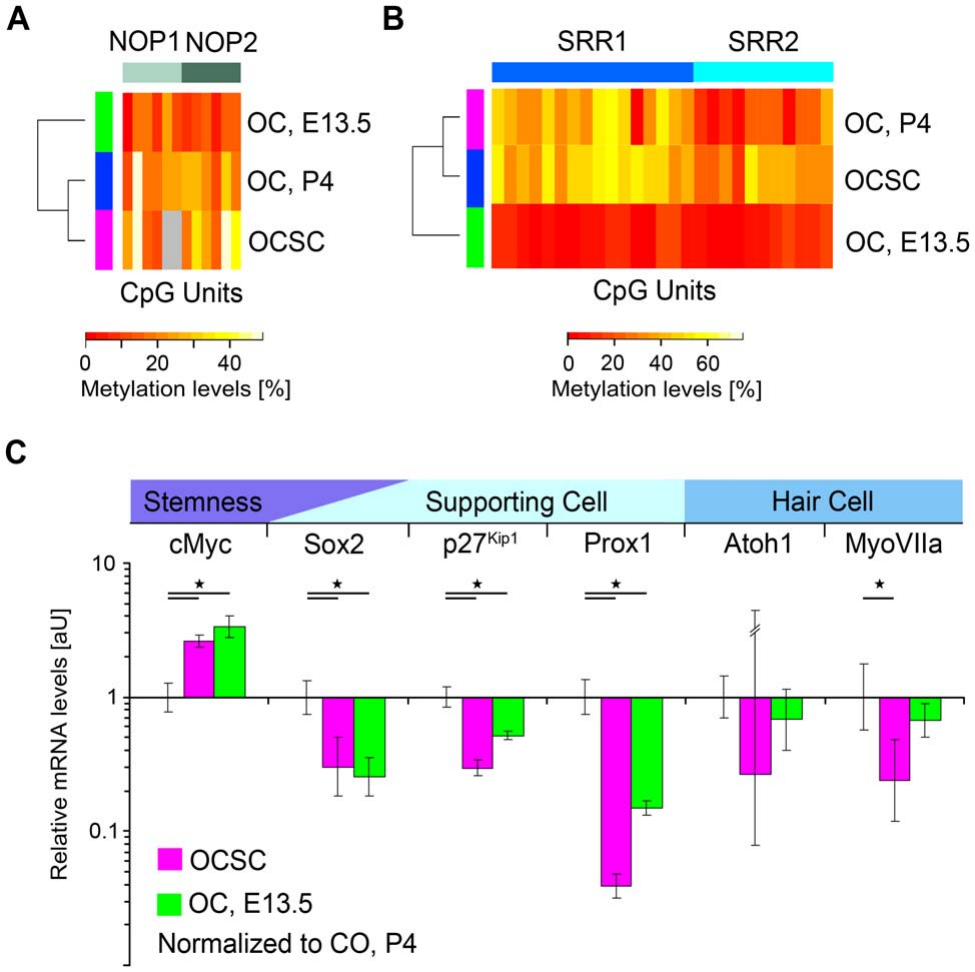
Epigenetic and transcriptional characterization of Sox2 during OCSC isolation. (**A**,**B**,**C**) Methylation profile of the Sox2 enhancers (**A**) SRR1/2, (**B**) NOP1 and (**C**) NOP2 in OCSCs as compared to the OC at P4 and E13.5 (i.e., see also Figure S4). (**D**) qPCR analysis of six developmentally regulated genes (cMyc, Sox2, Atoh1, myosin (Myo) VIIa, p27Kip1 and Prox1) in OCSCs and the OC at E13.5 and P4. Relative expression levels of OCSCs and E13.5 OC were compared with those of P4 OC. Transcript levels were normalized to HPRT1/Ubiquitin C levels. Averages of three independent experiments with SDs are depicted (*p<0.05) (i.e., see also Table S2C).

OCSCs exhibited no significant change in the methylation of the activated Sox2 promoter when compared with the P4 stage cells from which the otic spheres were isolated (Figure S4A). The methylation patterns of the silenced Nanog and Oct4 promoters also remained unaffected by the otic sphere formation procedure (Figure S4A). Furthermore, the CpG methylation pattern of the otic placode-related Sox2 enhancers NOP1 and NOP2 remained stable after otic sphere formation (P4: NOP1, 24%; NOP2, 23%; OCSC: NOP1, 25%; NOP2, 29%) (Figure 3A). Accordingly, cluster analysis revealed that no reprogramming of NOP1 or NOP2 was induced by otic sphere formation (Figure 3A). In addition, the methylation pattern of the ESC- and NSC-related Sox2 enhancer elements SRR1 and SRR2 remained unchanged (Figure 3B).

Sox2 mRNA expression was assessed using qPCR to determine whether otic sphere formation was accompanied by relative changes in Sox2 mRNA expression. To facilitate the comparative analysis, the data were normalized to the P4 developmental stage from which the OCSCs were isolated; this normalization allowed for a direct comparison of the OCSCs and the developmental progenitor stage at E13.5.

The OCSCs demonstrated a significant down-regulation of the transition gene Sox2 (p<0.01, Table S2C) as compared to P4, reaching levels similar to those at the proliferating progenitor stage at E13.5. This response correlated with a significant up-regulation of the early gene cMyc (p<0.01, Table S2C) and a concomitant, significant down-regulation of genes indicative of hair cell and supporting cell differentiation (p<0.01, Figure 3C, Table S2C).

To further explore and verify this transcriptional dedifferentiation response at the translational level, an immunohistochemical analysis was performed for Sox2 and other stemness markers. A comprehensive set of markers was analyzed at the three representative developmental time points (E13.5, P4 and P21) to distinguish Sox2-positive, proliferating otic progenitor cells (E13.5) from Sox2-positive, postmitotic supporting cells (P4 and P21).

**Figure 4:**
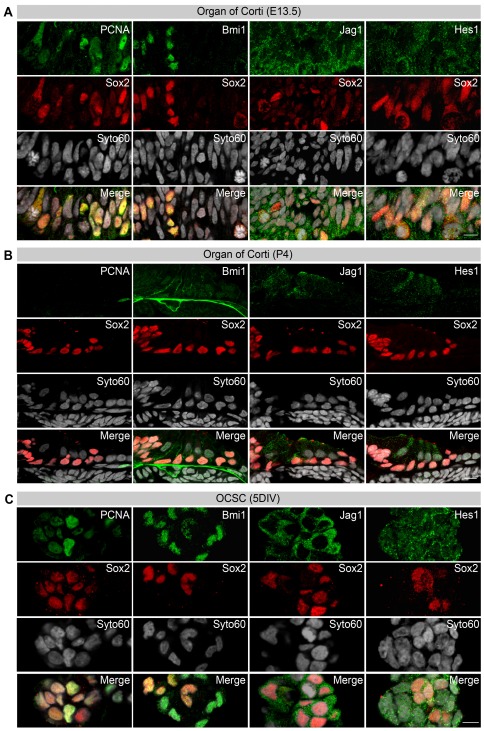
Characterization of Sox2 translation during OCSC isolation. (**A**,**B**) Double-labeling of Sox2 with PCNA, Bmi1, Jag1 and Hes1 in the OC at E13.5 and P4 compared to OCSCs. (**A**) Representative immunostaining images of longitudinal cryosections of the prosensory domain in the proximal cochlea duct at E13.5 (basilar membrane on top, luminal surface on the bottom). (**B**) Immature (P4) OC in mid-modiolar sections of the basal cochlea turn (medial to the left). (**C**) P4 OC-derived otic spheres after 5 DIV. Due to the requirements for the different tissue types investigated, the fixation, staining protocols and image acquisition settings were not identical (Scale Bars: A,B,C, 10 µm) (i.e., see also Figure S5).

The Sox2-positive progenitors (Figure 2A and 4A) differed in various aspects from quiescent (P4) (Figure 2B and 4B) and terminally differentiated (P21) Sox2-positive supporting cells (Figure 2C and S5A). First, co-localization of Sox2 with the proliferation markers Ki-67 (Figure 2A) or PCNA (Figure 4A) was only detected during the progenitor stage at E13.5 but never during the postnatal stages at P4 (Figure 4B and S5B) and P21 (Figure S5A). Second, when the expression of the adult stem cell markers Bmi1 [39] and Hmga2 [40], which are transcription factors known to antagonize p16^Ink4a^-mediated replicative senescence in NSC populations [29], was monitored during the OC development, an inverse relationship between p16^Ink4a^ and Bmi1 expression was observed. Pronounced Sox2/Bmi1 double labeling was characteristic of the otic progenitor stage at E13.5 (Figure 4A), but Bmi1 expression declined in the Sox2-expressing supporting cell domain of the OC at P4 (Figure 4B). At the same time point, an initial weak signal for p16^Ink4a^ was detected in a subset of Sox2-positive supporting cells (Figure S5B). At P21, the functionally mature stage, Sox2-positive supporting cells were completely devoid of Bmi1 expression (Figure S5A) but showed a strong signal for p16^Ink4a^ (Figure 2C). Hmga2 expression was found at all three developmental stages (Figure S5A, B, C) and in the OCSCs (Figure S5D). Third, activated Notch signaling was monitored by labeling the Notch ligand Jag1 and the Notch mediator Hes1. Both factors were strongly expressed in Sox2-positive progenitors at E13.5 (Figure 4A) but they became down-regulated as development proceeded; however, weak staining was maintained through P4 (Figure 4B) and P21 (Figure S5A).

The quiescent OC developmental stage at P4 gave rise to otic spheres with proliferating Sox2-positive cells, as shown by the co-localization of Sox2 with Ki-67 (Figure S5D) and PCNA (Figure 4C). In addition, 2-hour pulse-labeling of otic spheres with EdU indicated that 39±28% (n = 3, 12 spheres in total) of Sox2-positive cells were in S-phase, a sign of active proliferation (Figure S5D). In otic spheres, Sox2-positive cells also demonstrated double labeling with the stemness-related gene Bmi1 (Figure 4C) similar to E13.5 (Figure 4A). Furthermore, the Notch signaling markers Jag1 and Hes1 were labeled in otic spheres (Figure 4C) similar to the progenitor stage at E13.5 (Figure 4A). Taken together, dissecting the OC and applying defined *in vitro* culture conditions induced sphere formation. Sphere isolation itself had no effect on the epigenetic regulation of Sox2 in terms of a reprogramming response. However, the same assay accounted for a dedifferentiation response in the otic spheres, which became evident by the transcriptional regulation of Sox2 itself together with the mRNA and protein regulation of proliferation markers, stemness and Notch signaling markers in Sox2 positive cells.

### Otosphere differentiation is correlated with sequence-specific methylation of the enhancers NOP1 and NOP2

To confirm that the progressive developmental methylation of the enhancers NOP1 and NOP2 and the concomitant loss of cellular plasticity as seen in the fully differentiated OC at P21 was related to progressive differentiation, the experimental setup was inverted by applying differentiation-inducing culture conditions to the OCSCs after their formation. When dedifferentiated otospheres were transferred from suspension to adherent culture conditions, including growth factor withdrawal, the otospheres formed E-cadherin-positive, differentiating epithelial islands (Figure S6A). To evaluate the general differentiation potential of these epithelial islands, we analyzed the epigenetic, transcriptional and translational regulation of Sox2 expression in the context of other developmentally regulated genes. To compare *in vivo* and *in vitro* differentiation conditions, the OCSC stage after five days *in vitro* culture (5 DIV) served as the starting point. The fully differentiated OC at P21 *in vivo* was compared to epithelial island under differentiating culture conditions after 28 DIV roughly corresponding to the developmental time stretch from E13.5 to P21.

**Figure 5:**
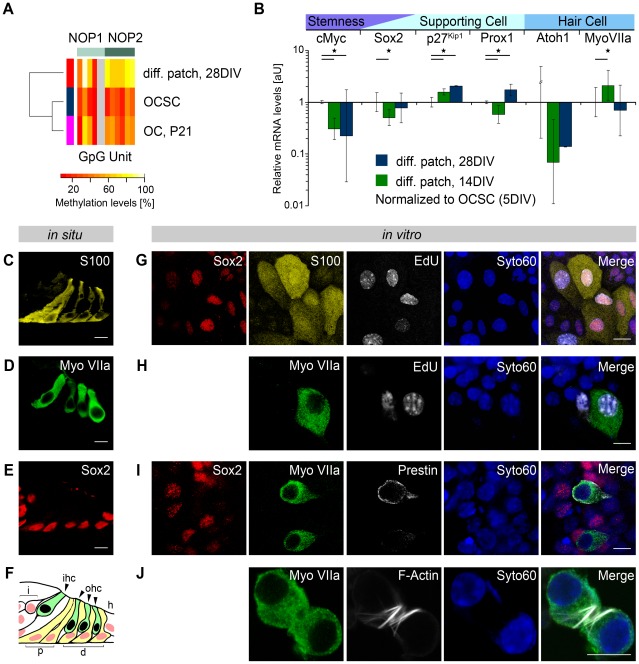
Differentiation potential of OCSCs. (**A**,**B**) Methylation profiles of the otic Sox2 enhancers (**A**) NOP1 and (**B**) NOP2 in the mature OC (P21), proliferating OCSC spheres and epithelial patches differentiated from OCSC spheres (i.e., see also Figure S4). (**C**) Relative expression levels of six developmentally regulated genes (cMyc, Sox2, Atoh1, myosin (Myo) VIIa, p27Kip1 and Prox1) after 14 and 28 days of differentiation (n = 3) were compared with those of the proliferating OCSC spheres by qPCR. Transcript levels were normalized to TbP/Ubiquitin C levels. Shown are averages of three independent experiments (and two independent experiments for 28 days for the differentiation group) with SDs (*p<0.05) (i.e., see also Table S2D). (**D**–**G**) *In situ* cell type-specific marker expression of the maturing OC (P4): Sox2 antibody (**F**) labels all supporting cells of the sensory domain (**G**), whereas S100-antibody (**D**) detects pillar and Deiters' cells only (**G**). Myosin VIIa (**E**) expression is associated with inner and outer hair cells (**G**). (**H**–**K**) OCSC-derived progeny differentiated under *in vitro* culture conditions. OCSC progeny were labeled by an EdU pulse (during the last day of 5 DIV) under proliferative culture conditions and a pulse chase after 14 DIV under differentiation-inducing culture conditions. EdU-labeling in supporting cell (Sox2, S100) (**H**) and hair cell-like (myosin VIIa) (**I**) cells. Hair cell-like cells were additionally characterized based on membrane-localized prestin (**J**) and F-actin-stained (**K**) membrane protrusions (Scale Bars: D,E,F,H,I,J,K, 10 µm) (i.e., see also Figure S6).

Under *in vitro* differentiation conditions, the Sox2 promoter remained constitutively active in the epithelial islands after 28 DIV (Figure S4A). However, the Sox2 enhancers NOP1 and NOP2 became silenced (NOP1, 56%; NOP2, 72%) (Figure 5A). The Oct4 and Nanog promoters remained silenced during *in vitro* and *in vivo* differentiation (Figure S4A).

Transcriptional aspects of differentiation in the epithelial islands were analyzed by qPCR after 14 and 28 DIV in differentiation culture conditions and normalized to the levels in otic spheres (5 DIV).

Although Sox2 has been classified as a transition gene during development (Figure 2E), Sox2 expression levels significantly declined (p<0.01) in the epithelial patches after 14 DIV but returned to OCSC levels at 28 DIV (Figure 5B, Table S2D).

To completely characterize the differentiation potential of OCSCs, we analyzed protein expression in epithelial patches at the single cell level by immunocytochemistry after 14 DIV and compared the patterns to the corresponding P4 developmental time point. At P4, myosin VIIa is a marker of early hair cell differentiation (Figure 5D), whereas Sox2 (Figure 5E) and S100 (Figure 5C) are expressed in a subset of cochlear supporting cells (Pillar and Deiters' cells) (Figure 5F). A 24-hour pulse of EdU at the end of the otic sphere culture period stably labeled the progeny of proliferating OCSCs. The fates of these EdU-labeled cells were tracked after 14 DIV in differentiating culture conditions. Some EdU-labeled cells differentiated into supporting and hair cell lineages. Newly generated Pillar- and Deiters' cell-like cells were indicated by the co-localization of EdU, Sox2 and S100 (Figure 5G). Hair cell-like cells were tracked by the co-localization of EdU and myosin VIIa (Figure 5H). Similar to the native OC (Figure 5F), organ-like cell clusters were detected. Myosin VIIa-positive hair cell-like cells appeared in close proximity to the Sox2-positive supporting cell-like cells (Figure 5I). Ongoing hair cell differentiation was further indicated by labeling with additional hair cell markers including myosin VI, parvalbumin and calretinin (Figure S6B). This hair cell differentiation progressed to the advanced stages, as indicated by labeling of the outer hair cell (OHC) marker protein prestin [41] (Figure 5I). At the subcellular level, expression of the membrane-bound protein prestin implies a progression of *in vitro* differentiation to the level of functional hair cells, which are normally found at the late developmental stage P12 [41]. This notion was further supported by the appearance of F-actin-positive stereocilia-like protrusions (Figure 5J) at the apical pole of the hair cell-like cells.

### EGF induces the sequence-specific methylation of NOP1 and NOP2 enhancers in parallel with a hollow sphere phenotype

**Figure 6:**
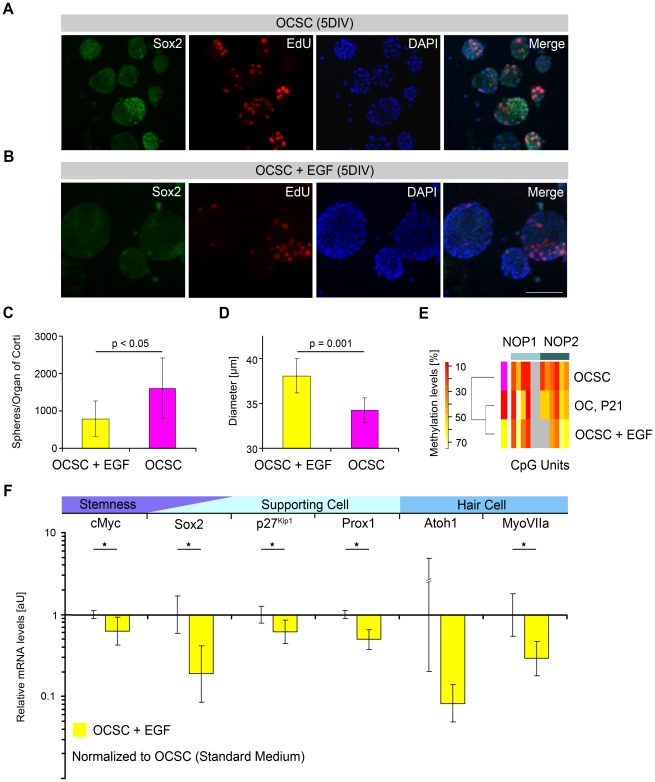
EGF interferes with the epigenetic regulation of Sox2 expression and affects the self-renewal potential of OCSCs. (**A**,**B**) P4 OC-derived otospheres after 5 DIV; labeling for Sox2 combined with EdU and DAPI (**A**) Otospheres grown under FGF/IGF-only conditions. (**B**) Otospheres supplemented with EGF as an additional growth factor (Scale Bars: A,B, 100 µm). (**C**) Absolute numbers of primary spheres isolated per OC with (n = 7) and without EGF (n = 8) supplementation. Data were analyzed by student's t-test and are presented as means ±SDs. (**D**) Mean diameter of the primary sphere population measured in a range from 25 to 60 µm with (n = 7) and without EGF (n = 8) supplementation. Data are presented as means ±SDs. (**E**) Methylation profiles of the otic Sox2 enhancers NOP1/2 in P21 OC, proliferating OCSCs and OCSCs supplemented with EGF (i.e., see also Figure S4). (**F**) qPCR analysis of six developmentally regulated genes (cMyc, Sox2, Atoh1, myosin VIIa, p27Kip and Prox1) in standard OCSCs and in OCSCs supplemented with EGF. Relative expression levels of standard OCSCs were compared to those of OCSCs supplemented with EGF. Transcript levels were normalized to HPRT1/TbP levels. Averages of three independent experiments are shown with SDs (*p<0.05) (i.e., see also Table S2E).

The increased methylation of NOP1 and NOP2 observed under differentiating culture conditions parallels the *in vivo* situation, raising the question of whether the experimental conditions can also be modified to promote the methylation of NOP1 and NOP2 under primary sphere forming conditions resulting in a concomitant loss of the stemness assigned to OCSCs. The observed dedifferentiation response to generate OCSCs required a specific combination of cell culture medium, supplements and growth factors. Systematic variation in the composition of the growth medium was used to assess otic sphere formation capacity. Since the self-renewal potential of the total otic sphere population has previously been ascribed to the solid fraction of otic spheres [27], appearance of different sphere phenotypes has been investigated. The standard growth factor combination used in this investigation consisted of FGFb (10 ng/ml), IGF1 (50 ng/ml) and heparin sulfate (HS) (2 µg/ml) (Figure 6A). The addition of a third factor, EGF (20 ng/ml) (Figure 6B), resulted in a significant decrease in the primary solid sphere fraction (Figure 6C), which was measured using a diameter range of 25 to 60 µm using objective, automated sphere counting. Under FGF/IGF-only culture conditions, a single OC gave rise to approximately 1606±810 otic spheres (Figure 6C) with a mean diameter of 34.2±1.4 µm (Figure 6D) (independent experiments: n = 8). Addition of EGF significantly reduced the number of otic spheres to 790±472 (p<0.05) (Figure 6C), whereas the mean diameter of the measured sphere population increased significantly to 38.1±1.9 µm (p = 0.001) (Figure 6D) (independent experiments: n = 7). The EGF induced increase in sphere diameter was accompanied by a sphere volume gain of about 38%. Morphological analysis revealed the EGF-dependent increase in volume as indicative for the switch from the solid/self-renewing to the hollow/non-self-renewing phenotype (Figure 6A,B). We thus sought to determine whether EGF signaling interferes with the epigenetic regulation of NOP1 and NOP2 in OCSCs.

The CpG methylation patterns of the Sox2 promoter and otic enhancers were analyzed with EGF as an additional growth factor; these patterns were compared to those in the FGF/IGF-only conditions and to the developmental data. EGF supplementation had no effect on the methylation of the Sox2, Nanog and Oct4 promoters (Figure S4A); however, methylation of the otic enhancers NOP1 and NOP2 increased in EGF-treated spheres as compared to OCSCs grown without EGF (NOP1: non-EGF, 24%; EGF, 36%; NOP2: non-EGF, 29%; EGF, 49%) (Figure 6E). Surprisingly, under EGF conditions, the NOP1 and NOP2 enhancer methylation status did not resemble that of the progenitor stage at E13.5 or of the otic sphere under FGF/IGF-only conditions but instead clustered with that of the functionally mature OC (P21). Therefore, the methylation pattern of NOP1 and NOP2 under EGF conditions was similar to the pattern observed in the functionally mature OC under *in vivo* conditions. Additionally, the assessed mRNA profiles differed from those observed in otic spheres grown with FGF/IGF alone. The relative Sox2 mRNA expression level was also significantly reduced (p<0.01) as compared with its basal expression level in OCSCs grown under FGF/IGF-only conditions (Figure 6F, Table S2E).

These results suggest that non-cell-autonomous factors like EGF supplementation induce NOP1- and NOP2-specific methylation under otic sphere-formation conditions, causing a concomitant down-regulation of Sox2 expression. The conversion of the otic sphere phenotype from a solid, self-renewing type under non-EGF conditions to a hollow, non-self-renewing type under EGF-treatment starkly supports the interconnection between NOP1 and NOP2 methylation and a loss of stemness.

## Discussion

**Figure 7:**
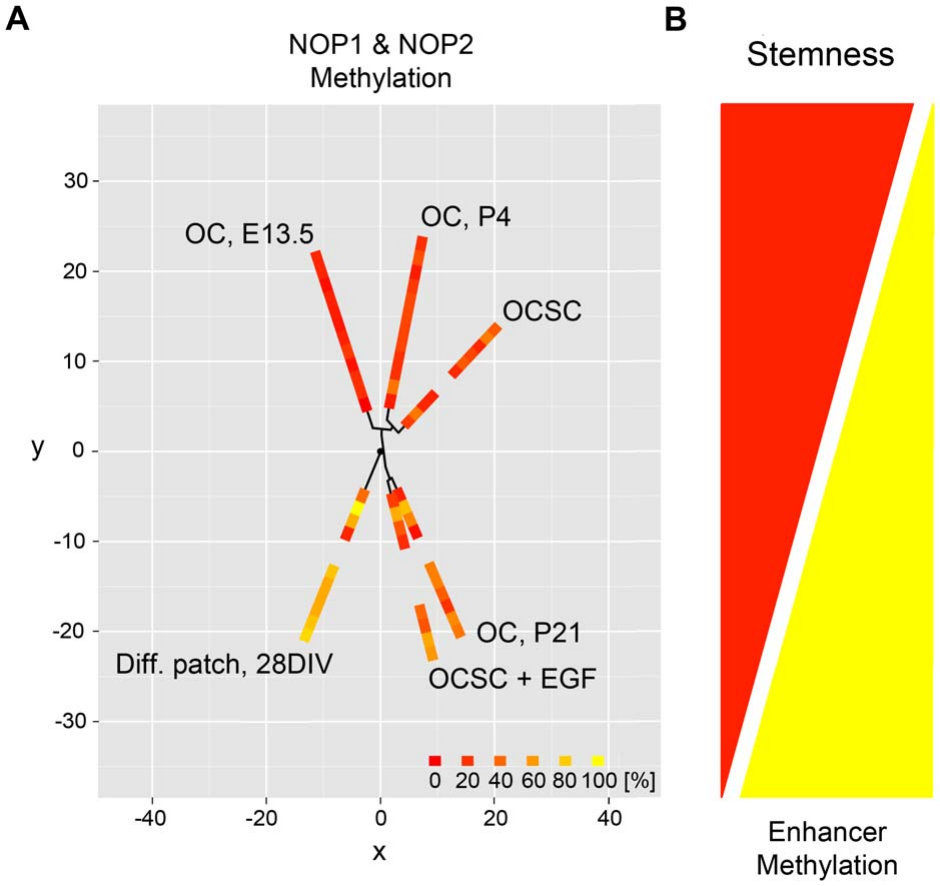
Methylation patterns of the otic Sox2 enhancers NOP1 and NOP2 are differentially regulated with regard to stemness and otic differentiation. (**A**,**B**) The complete NOP1/2 datasets were analyzed by a circular map to visualize the relationships between analyzed elements with respect to the topology inherent in the data. NOP1 and NOP2 methylation levels of OC (E13.5, P4 and P21) as compared to OCSC, OCSC+EGF and differentiating epithelial island (28 DIV). The map is similar to clustering, but the arrangement is circular rather than linear to emphasize the periodicity of the angular positions and to allow comparisons across conditions and factors.

The findings presented in this study indicate that a low or moderate methylation status of the tissue-specific Sox2 enhancers NOP1 and NOP2 was correlated with a permissive role of Sox2 with regards to otic stemness, as seen in OCSCs and embryonic progenitors or stem cell isolation potential as seen in postnatal supporting cells. In contrast, progressive methylation was related to supporting cell differentiation and loss of stemness both *in vitro* and *in vivo* as well as in response to EGF treatment. Integration of NOP1 and NOP2 methylation data into a comprehensive circular map allowed visualization of these relationships showing the association of the demethylated status with stemness above the y-axis and the methylated status with a loss of stemness below the y-axis (Figure 7A). This suggests that the methylation status of the otic Sox2 enhancers NOP1 and NOP2 are inversely related to conditions permissive to otic stemness (Figure 7B).

We generated OCSCs using an otic sphere forming assay and then compared the molecular signatures of these OCSCs to those of ESCs and NSCs. Specifically, levels of the three critical transcription factors that establish the pluripotency network, Oct4, Nanog and Sox2, were measured [42]. Oct4 and Nanog were down-regulated in OCSCs at the epigenetic, transcriptional and translational level, which was also observed in multipotent NSCs but not in ESCs. Similarly, in inner ear stem cells derived from the utricle Sox2 expression is maintained, while Nanog and Oct4 transcription is down-regulated [43]. Therefore, OCSCs show no pluripotent developmental potential at the molecular level. However, in NSCs single factor reprogramming by Oct4 in addition to endogenously expressed Sox2 is sufficient for acquiring pluripotency [44]. Therefore, based on endogenous Sox2 expression OCSCs may also be amendable to Oct4 single factor reprogramming.

Similar to NSCs the expression pattern of Sox2 in OCSCs suggests that Sox2 plays a key role in the observed multipotency of OCSCs. As a differentiating feature, at the Sox2 enhancer level, the increased methylation status for SRR1 and SRR2 in OCSCs is distinct from that of NSCs and ESCs; this finding suggests that in OCSCs Sox2 expression is controlled by the otic enhancers NOP1 and NOP2 [25]. A previous investigation of the evolutionally conserved Sox2 enhancers SRR1 and SRR2 [45] in human NT2-D1 cells revealed a cell fate-specific methylation pattern in the regulatory element SRR2. A different role was found for SRR1, indicating a correlation between methylation state and proliferative potential. Such a functional division was not evident in our investigation of the otic enhancers NOP1 and NOP2, as their methylation patterns appeared uniform. Since supporting cell types are overrepresented in cochlear tissue and *in vitro* culture preparations, the discussion of the results reported here relates to supporting and stem/progenitor cell fates.

During embryonic development of the inner ear Sox2 expression is controlled by inner ear-selective enhancer elements and represents a critical factor in establishing the prosensory domain of the OC, as previously demonstrated in two allelic Sox2 mouse mutants, *Lcc* and *Ysb* [18]. Because neither the protein-coding region nor the promoter of Sox2 was affected by these mutations [18], these mutants directly indicate the key role of the tissue-specific enhancer elements NOP1 and NOP2, previously identified as enhancers in the avian inner ear [25], in regulating Sox2 expression in the mammalian OC. Sequence analysis reported in this study revealed that the evolutionarily conserved avian NOP1 and NOP2 sequences map to the murine genomic locus affected by the *Lcc* mutation. Although the two integration sites of the *Ysb* mutants did not alter the wild-type NOP1 and NOP2 sequences, the identified chromosomal rearrangement might interfere [18]. These findings imply that the *Lcc* and *Ysb* phenotypes are correlated with a compromise in function of the murine NOP1 and NOP2 enhancer elements.

It has been suggested that Sox2 plays a dose-dependent role in the inversely correlated phenomena of stemness and differentiation [19]. Further evidence for a Sox2 rheostat-like function is provided by the present study, which revealed that Sox2 mRNA levels change during development. At the same time that Sox2 levels increase from embryonic (E13.5) to postnatal (P4) time points, an auto-regulatory loop mediated by a Sox2 binding site in the NOP1 and NOP2 enhancer elements might be activated during early postnatal development. This relationship possibly contributes to the maintenance of the supporting cell phenotype, which would be consistent with the previously reported finding of a reciprocal antagonistic relationship between Sox2 in the differentiating supporting cells and Atoh1 in the differentiating hair cells [19]. During late postnatal development, Sox2 transcriptional down-regulation is correlated with an increase in NOP1 and NOP2 enhancer methylation in the functionally mature OC at P21. *Vice versa* at the embryonic developmental stage low Sox2 levels may be related to early Notch signaling. Indeed, conditional gene targeting has previously identified Sox2 as a target gene of the Notch ligand Jag1, which is strongly expressed in the proliferating primordium before cell differentiation [46]. In the inner ear, Notch signaling is mediated by the bHLH transcription factor Hes1 [47]. The bHLH transcription factors are known to interact with E-box motifs [48], which have been computationally identified in the NOP1 and NOP2 sequences in our study. As the bHLH transcription factors act as transcriptional repressors, the binding of Hes1 to NOP1 and NOP2 could account for the maintenance of Sox2 expression at the low basal level found in the embryonic OC. Consistent with this hypothesis, Hes1 expression is down-regulated in the nascent OC by E14.5 [47], when the cells exit the cell cycle and undergo cell fate decisions towards hair cell and supporting cell phenotypes. Therefore, the early Notch signaling-induced transcriptional silencing of NOP1 and NOP2 may be released and result in an up-regulation of Sox2 expression, thus contributing to the differentiation and maintenance of supporting cells.

Our results did not reveal any sequence-specific demethylation of the investigated promoters or Sox2 enhancer elements induced by the otic sphere assay. Therefore, OCSC formation in the otic sphere assay may reflect an *in vitro* dedifferentiation response of the postmitotic supporting cells rather than a reprogramming of the somatic cells into stem cells after tissue explantation and dissociation [49].

Nevertheless, the observed regulation of methylation patterns in the tissue-specific Sox2 enhancer elements NOP1 and NOP2 points to a key role for epigenetic mechanisms in determining the regenerative potential of the OC. In this study, we demonstrated that the NOP1 and NOP2 enhancers have a demethylated status at the developmental stage of the otic progenitors at E13.5. The progressive developmental methylation of both enhancers is correlated with a loss of stem cell isolation capacity in the functionally mature developmental stage.

The Sox2 enhancer methylation levels in otic cells appear to depend on non-cell autonomous factors, as demonstrated by the effect of exogenous EGF application. The observed interplay between EGF signaling and a reduced phenotypic self-renewal potential has also been shown for NSCs in the subventricular zone (SVZ) [50]. In that study, infusion of EGF into the lateral ventricle of mice led to the proliferation of EGFR-expressing neural progenitor cells. However, the potential for proliferation and self-renewal of NSCs from the EGF-infused SVZ was reduced as compared with controls. Thus, our finding of an EGF-induced hollow otic sphere phenotype with a reduced self-renewal potential [27] and the reduced self-renewal potential of NSCs of the SVZ [50] indicate that self-renewal potential in both multipotent stem cell types is negatively regulated by EGF supplementation. In addition, EGF has been also shown to induce differentiation of cochlear hair cells from dividing progenitor cells from the embryonic developmental stage E13.5 which were directly isolated and plated as epithelial island without going through sphere formation [51].

It has been previously reported that otic sphere formation is related to a gain in developmental potential, with transcriptional and translational changes that are indicative of dedifferentiation [2], [4]. The results of this study further support these findings for the OC. Dedifferentiation as seen in otosphere formation evokes a phenotypic switch and a transcriptional and translational conversion from postmitotic supporting cells into proliferating primordial-like cells by a dynamic regulation of the transcriptome.

Future hair cell regeneration strategies should consider supporting cell reprogramming to render senescent supporting cells responsive to dedifferentiation. To a limited extent, spontaneous dedifferentiation responses have been observed in the OC after hair cell damage (e.g., reactivation of embryonic Notch signaling) [52]. We speculate that the addition of small molecule-based reprogramming or dedifferentiation factors to cochlear supporting cells could be a reasonable strategy for reactivating the cells' endogenous regenerative potential, thereby allowing epimorphic hair cell regeneration.

## Materials and Methods

### Animals

All mice used in this study were C57/BL6 background (Charles River Laboratories, Sulzfeld, Germany); breeding and maintenance were performed in an in-house animal facility. The use of animals for organ explantation and stem cell isolation was reviewed and approved by the Committee for Animal Experiments of the Regional Council (Regierungspräsidium) of Tübingen.

### Inner ear dissection

Mice were used at embryonic day (E) 13.5 and postnatal days (P) 4 and P21. Mice were then euthanized with CO_2_ and decapitated. After removing the brain, the inner ear bony labyrinth capsules were dissected from the skull base in Hank's buffered saline solution (HBSS). Fixation of the inner ear was carried out by perfusion of the oval window, the round windows and an additional hole in the apex of the cochlea with 4% PFA. Only P21 inner ears were decalcified with 0.2 M EDTA in PBS for 2 days before being sliced into cryosections. After incubation in sucrose (30% in PBS), preparations were embedded in a Tissue-Tek® OCT™ Compound (Sakura Finetek, Zoeterwoude, The Netherlands) and stored at −80°C. Cryosectioning was performed with a Microm cryostat (Microm Laborgeräte GmbH, Walldorf, Germany).

### Immunohistochemistry

After blocking with 1% BSA in 0.2% Triton PBS, cryosections were incubated overnight at 4°C with primary antibodies (Table S3). After washing with 0.2% Triton/PBS, primary antibodies were detected using Alexa-conjugated secondary antibodies for 60 min at RT (Table S3). The sections were counterstained with DAPI, Syto60 or Sytox Green (Molecular Probes–Invitrogen, Darmstadt, Germany) and mounted with FluorSave™ (Calbiochem-Merck, Darmstadt, Germany). The sections were analyzed using a Zeiss 510 Meta confocal laser-scanning microscope (Zeiss, Göttingen, Germany).

### Cell culture

ESCs, NSCs and OCSCs were from a C57/BL6 mouse background. Details and culture procedures, including otosphere isolation and otic differentiation, are described in the Supplemental Experimental Procedures S1.

### Bisulfite conversion/Quantitive methylation analysis (EpiTyper) and real-time PCR

Inner ear tissues and cultured cells from 24-well tissue culture plates (Greiner 35/10) were isolated and immediately frozen in liquid nitrogen prior to lysis for RNA or gDNA isolation. Details are available in the Supplemental Experimental Procedures S1.

### Chicken, human and mouse *Sox2* sequence analysis

Sequences were analyzed with Genomatix DiAlign professional Release 3.1.4 software (Genomatix Software GmbH, Munich, Germany). DNA motifs were scanned using YEASTRACT-DISCOVERER software (http://www.yeastract.com/cite.php).

## Supporting Information

Figure S1 **NOP1 and NOP2 nucleotide sequences.** (**a**, **b**) Nucleotide sequences of the otic enhancers NOP1 and NOP2 of the chicken Sox2 locus and alignment with the corresponding human and mouse sequences. Sequences are shaded where the nucleotide residue is conserved in all species. CpG sites are underlined, and putative binding sequences of various transcription factors conserved among the animal species are shown in boxes.
(TIF)

Figure S2 **NOP1 and NOP2 are covered by the Lcc locus and may potentially interfere with the Ysb locus.** (**a**) Ideogram of mouse chromosome 3 showing the putative Ysb and Lcc rearrangement sites. Blue and green bars, transgenes at insertion sites 1 and 2; red bar, putative inversion of *Lcc* (modified from Dong et al., 2002). (**b**) *Lcc* and *Ysb* wild type loci. Sox2 coding region (including promoter) and Sox2 enhancers NOP1/2 are covered by the Lcc locus as determined by linkage analysis of polymorphic microsatellite markers (modified from Dong et al., 2002). Due to the relative proximity to *Ysb* integration sites 1/2 determined in the wild-type sequence, NOP1/2 might also interfere with *Ysb* rearrangements (modified from Dong et al., 2002).
(TIF)

Figure S3 **Criteria used to assign developmentally regulated genes to early, transition or differentiation gene groups.** (**a**) Early genes were primarily expressed in the progenitor cell population at E13.5, with significant down-regulation at P4. (**b**) Transition genes were expressed up to P4, with significant down-regulation in the mature OC at P21. (**c**) Differentiation genes were expressed at very low levels in proliferating progenitors at E13.5, were significantly up-regulated at P4 and were stably maintained in the functional epithelium at P21. (**d**) The relative amount of each gene transcript was determined by qPCR assay, and data were analyzed using the ΔΔ C_T_ method. Primer efficiencies for unknown and reference genes were confirmed using standard curve experiments. The C_T_ value determines the cycle threshold when the fluorescence reading surpassed a set baseline. Depending on the C_T_ value, genes were classified as high and low copy number genes. C_T_ values <35 were classified as background.
(TIF)

Figure S4 **DNA methylation patterns during otic development** ***in situ*** **and** ***in vitro*** **.** (**a**) Bisulfite methylation profiles for Sox2, Nanog and Oct4 promoters in the OC at three developmental time points (E13.5, P4, P21), OCSCs (OCSC), EGF-treated OCSCs (OCSC+EGF) and differentiated epithelial patches (28 DIV). DNA methylation values are shown on a pseudo-color scale (methylation increases from red [non-methylated] to yellow [methylated]); missing values are shown in grey.
(TIF)

Figure S5 **Characterization of Sox2 translation.** (**a**–**c**) OC in mid-modiolar sections of the basal cochlear turn (medial to the left). (**a**) OC at P21; labeling for Sox2 combined with PCNA, Bmi1, Jag1, Hes1, Ki-67 and Hmga2. (**b**) OC at P4; labeling for Sox2 combined with p16Ink4a, Ki-67 and Hmga2. (**c**) OC at E13.5; labeling for Sox2 combined with Hmga2. (**d**) P4 OC-derived otospheres after 5 DIV; labeling for Sox2 combined with EdU, Ki-67 and Hmga2 (Scale Bars: **a**, **b**, **c**, **d**; 10 µm).
(TIF)

Figure S6 **Differentiation potential of OCSCs.** (**a**, **b**) OCSC-derived progeny differentiated under differentiating *in vitro* culture conditions (14 DIV). (**a**) E-cadherin-positive epithelial island containing supporting cell-like cells, labeled for S100 and Sox2 (**b**) Epithelial island containing hair cell-like cell, triple-labeled for parvalbumin, calretinin and myosin VI (Scale Bars: **a**, **b**, 10 µm).
(TIF)

Table S1 **NOP1 and NOP2 enhancers of the Sox2 gene and their conservation across chickens, mice and humans.**
(TIF)

Table S2 **qPCR data shown as ΔΔ C_T_ values.**
(XLS)

Table S3 **Antibodies and fluorophores used in the study.**
(TIF)

Supplementary Experimental Procedures S1 (DOC)
