# Supplementary figures and images for: Stemness of the Organ of Corti Relates to the Epigenetic Status of Sox2 Enhancers
Source: PLoS One. 2012 May 3;7(5):e36066. doi: 10.1371/journal.pone.0036066 (PMC3343037; doi:10.1371/journal.pone.0036066)

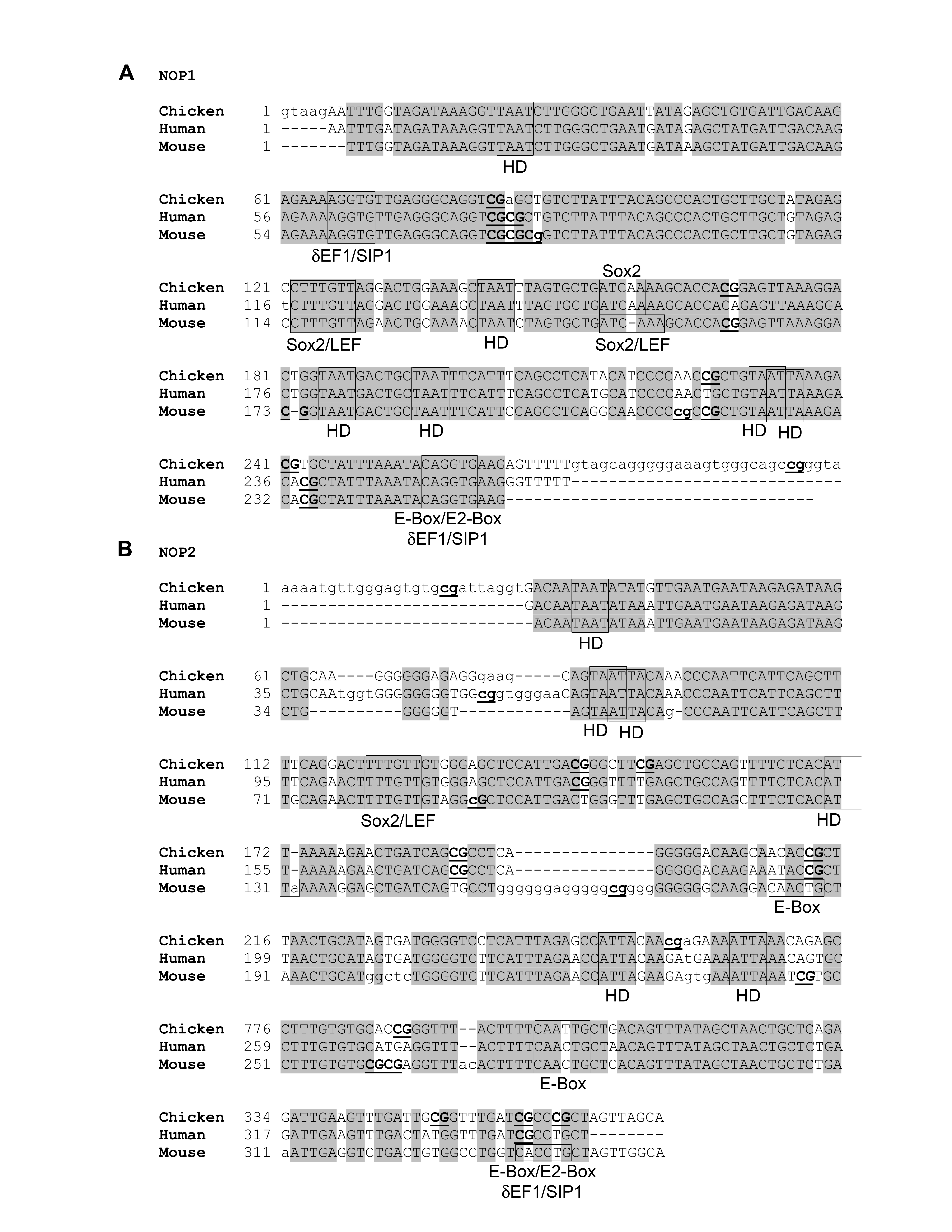

Supplement: Figure S1 — NOP1 and NOP2 nucleotide sequences. (a, b) Nucleotide sequences of the otic enhancers NOP1 and NOP2 of the chicken Sox2 locus and alignment with the corresponding human and mouse sequences. Sequences are shaded where the nucleotide residue is conserved in all species. CpG sites are underlined, and putative binding sequences of various transcription factors conserved among the animal species are shown in boxes. (TIF) [file pone.0036066.s001.tif]

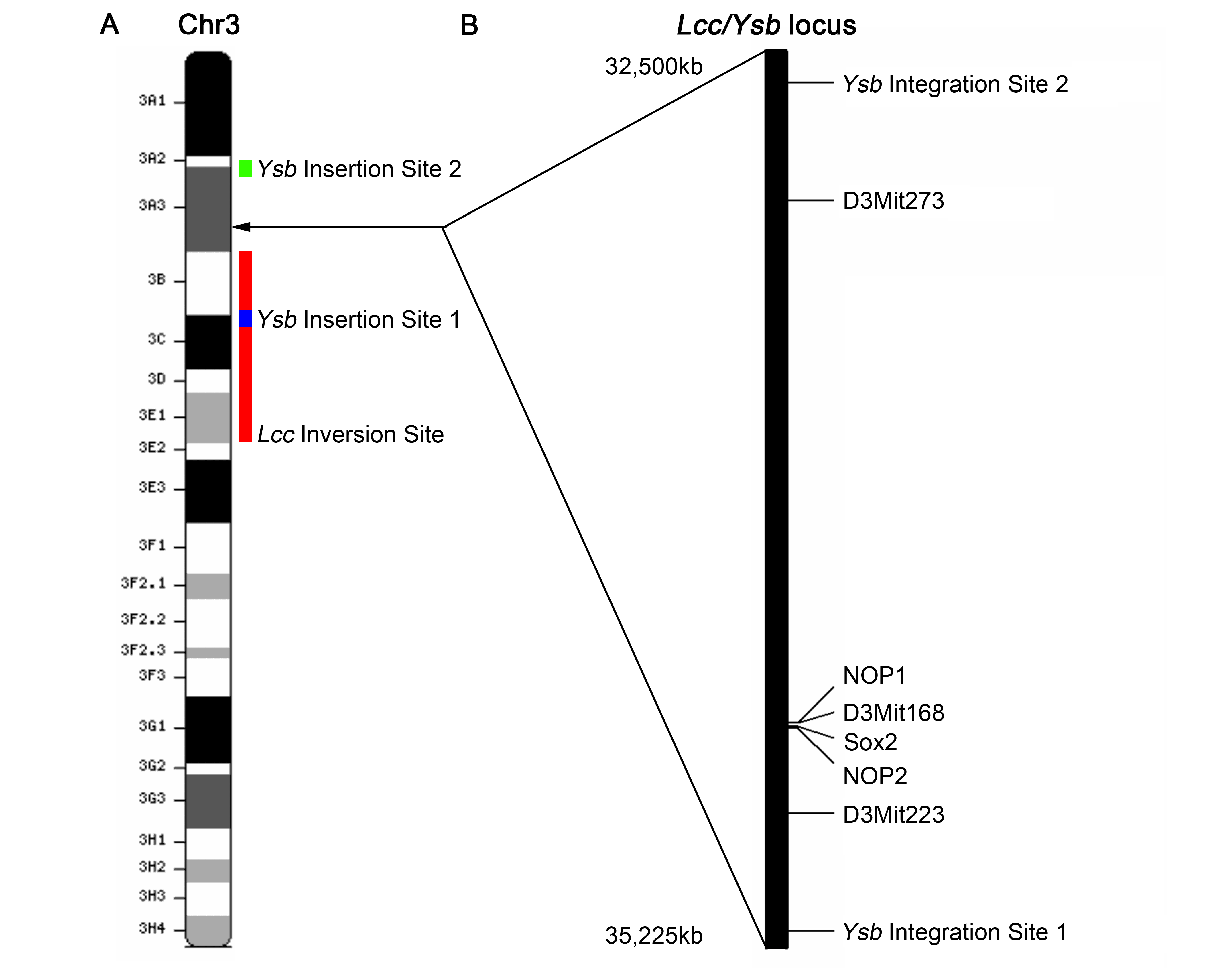

Supplement: Figure S2 — NOP1 and NOP2 are covered by the Lcc locus and may potentially interfere with the Ysb locus. (a) Ideogram of mouse chromosome 3 showing the putative Ysb and Lcc rearrangement sites. Blue and green bars, transgenes at insertion sites 1 and 2; red bar, putative inversion of Lcc (modified from Dong et al., 2002). (b) Lcc and Ysb wild type loci. Sox2 coding region (including promoter) and Sox2 enhancers NOP1/2 are covered by the Lcc locus as determined by linkage analysis of polymorphic microsatellite markers (modified from Dong et al., 2002). Due to the relative proximity to Ysb integration sites 1/2 determined in the wild-type sequence, NOP1/2 might also interfere with Ysb rearrangements (modified from Dong et al., 2002). (TIF) [file pone.0036066.s002.tif]

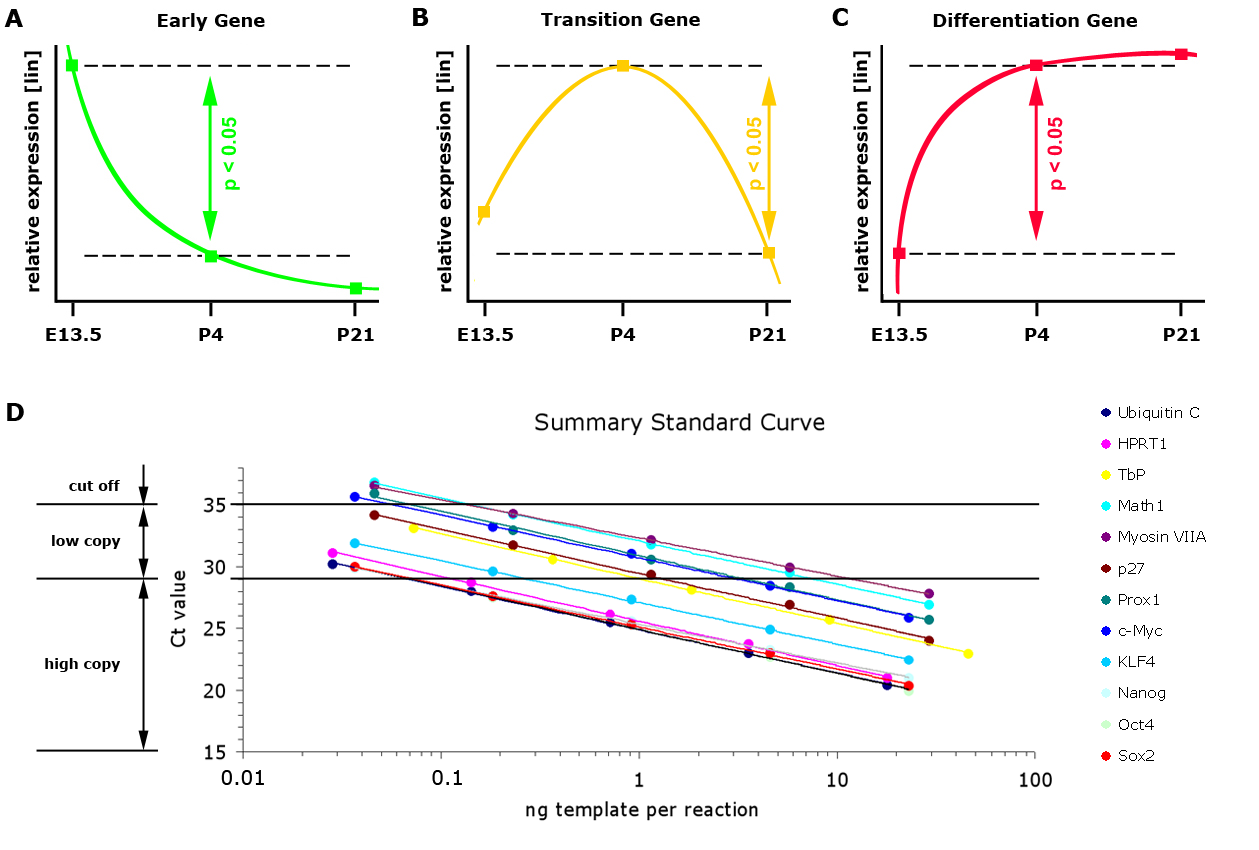

Supplement: Figure S3 — Criteria used to assign developmentally regulated genes to early, transition or differentiation gene groups. (a) Early genes were primarily expressed in the progenitor cell population at E13.5, with significant down-regulation at P4. (b) Transition genes were expressed up to P4, with significant down-regulation in the mature OC at P21. (c) Differentiation genes were expressed at very low levels in proliferating progenitors at E13.5, were significantly up-regulated at P4 and were stably maintained in the functional epithelium at P21. (d) The relative amount of each gene transcript was determined by qPCR assay, and data were analyzed using the ΔΔ CT method. Primer efficiencies for unknown and reference genes were confirmed using standard curve experiments. The CT value determines the cycle threshold when the fluorescence reading surpassed a set baseline. Depending on the CT value, genes were classified as high and low copy number genes. CT values <35 were classified as background. (TIF) [file pone.0036066.s003.tif]

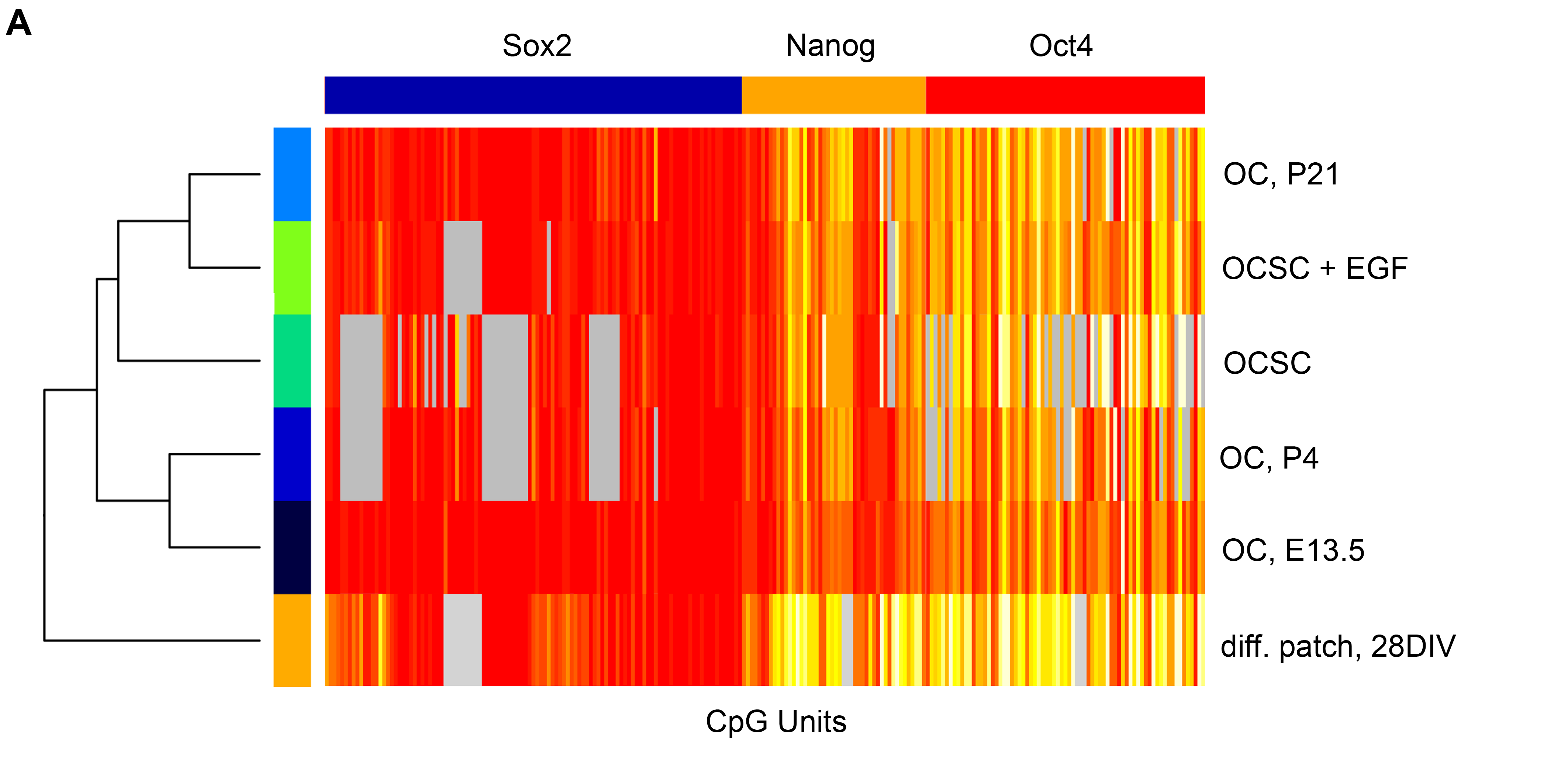

Supplement: Figure S4 — DNA methylation patterns during otic development in situ and in vitro . (a) Bisulfite methylation profiles for Sox2, Nanog and Oct4 promoters in the OC at three developmental time points (E13.5, P4, P21), OCSCs (OCSC), EGF-treated OCSCs (OCSC+EGF) and differentiated epithelial patches (28 DIV). DNA methylation values are shown on a pseudo-color scale (methylation increases from red [non-methylated] to yellow [methylated]); missing values are shown in grey. (TIF) [file pone.0036066.s004.tif]

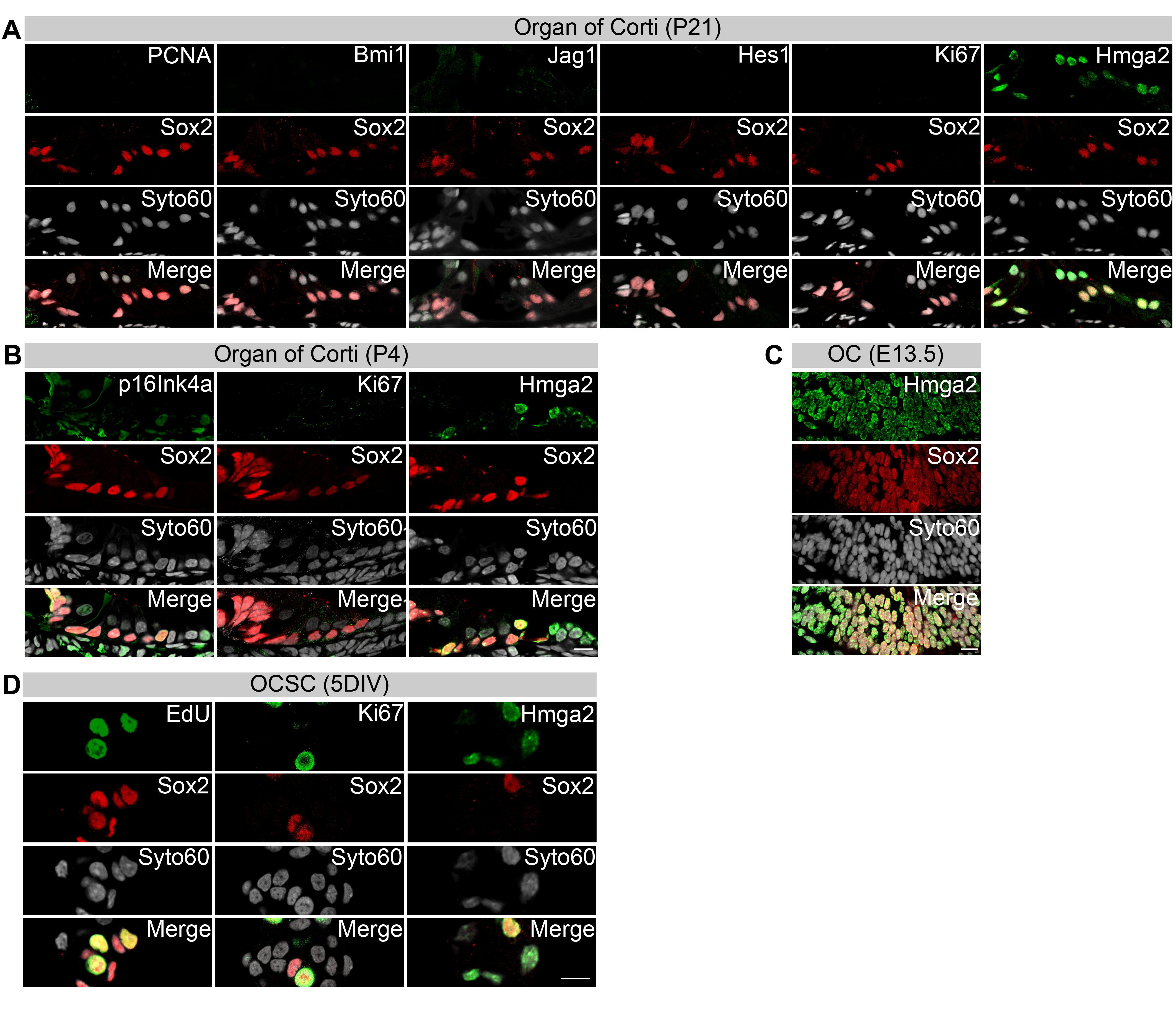

Supplement: Figure S5 — Characterization of Sox2 translation. (a–c) OC in mid-modiolar sections of the basal cochlear turn (medial to the left). (a) OC at P21; labeling for Sox2 combined with PCNA, Bmi1, Jag1, Hes1, Ki-67 and Hmga2. (b) OC at P4; labeling for Sox2 combined with p16Ink4a, Ki-67 and Hmga2. (c) OC at E13.5; labeling for Sox2 combined with Hmga2. (d) P4 OC-derived otospheres after 5 DIV; labeling for Sox2 combined with EdU, Ki-67 and Hmga2 (Scale Bars: a, b, c, d; 10 µm). (TIF) [file pone.0036066.s005.tif]

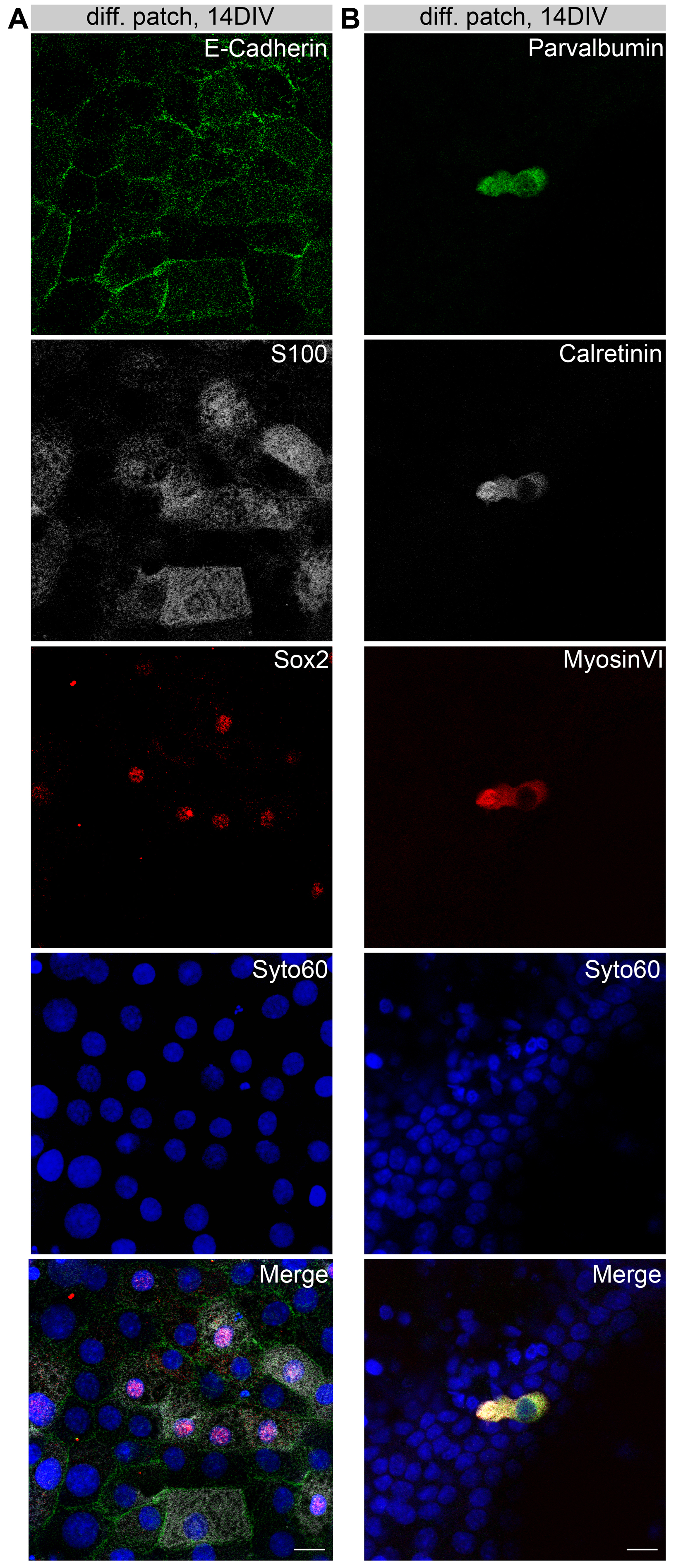

Supplement: Figure S6 — Differentiation potential of OCSCs. (a, b) OCSC-derived progeny differentiated under differentiating in vitro culture conditions (14 DIV). (a) E-cadherin-positive epithelial island containing supporting cell-like cells, labeled for S100 and Sox2 (b) Epithelial island containing hair cell-like cell, triple-labeled for parvalbumin, calretinin and myosin VI (Scale Bars: a, b, 10 µm). (TIF) [file pone.0036066.s006.tif]

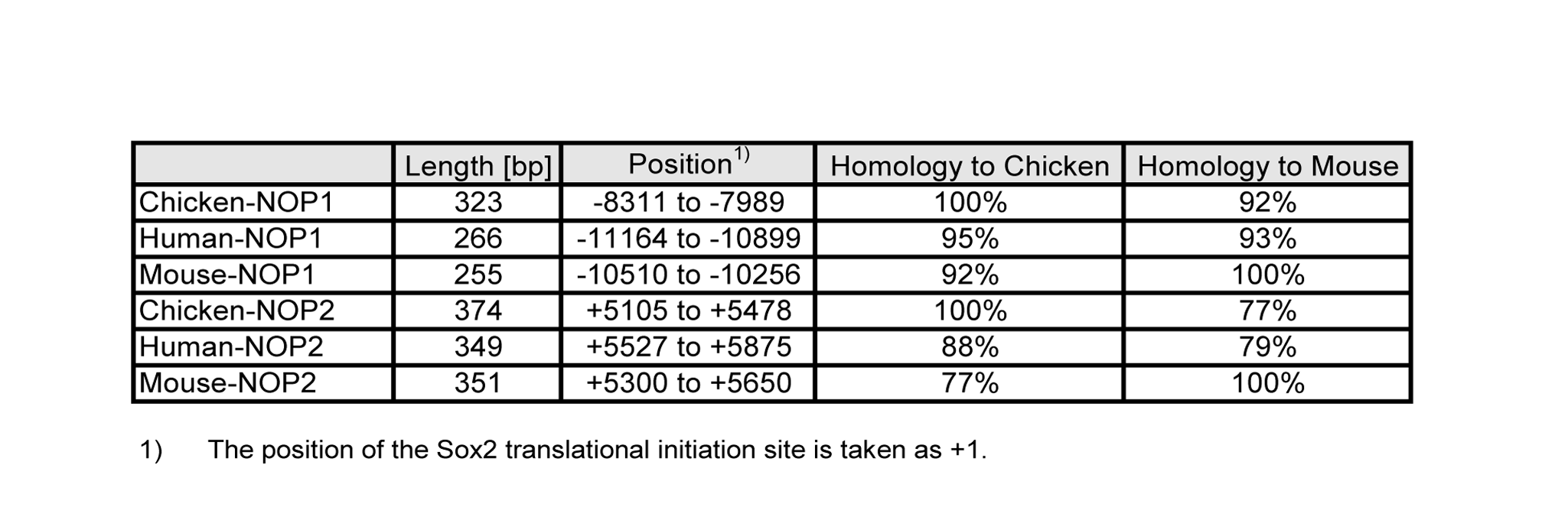

Supplement: Table S1 — NOP1 and NOP2 enhancers of the Sox2 gene and their conservation across chickens, mice and humans. (TIF) [file pone.0036066.s007.tif]

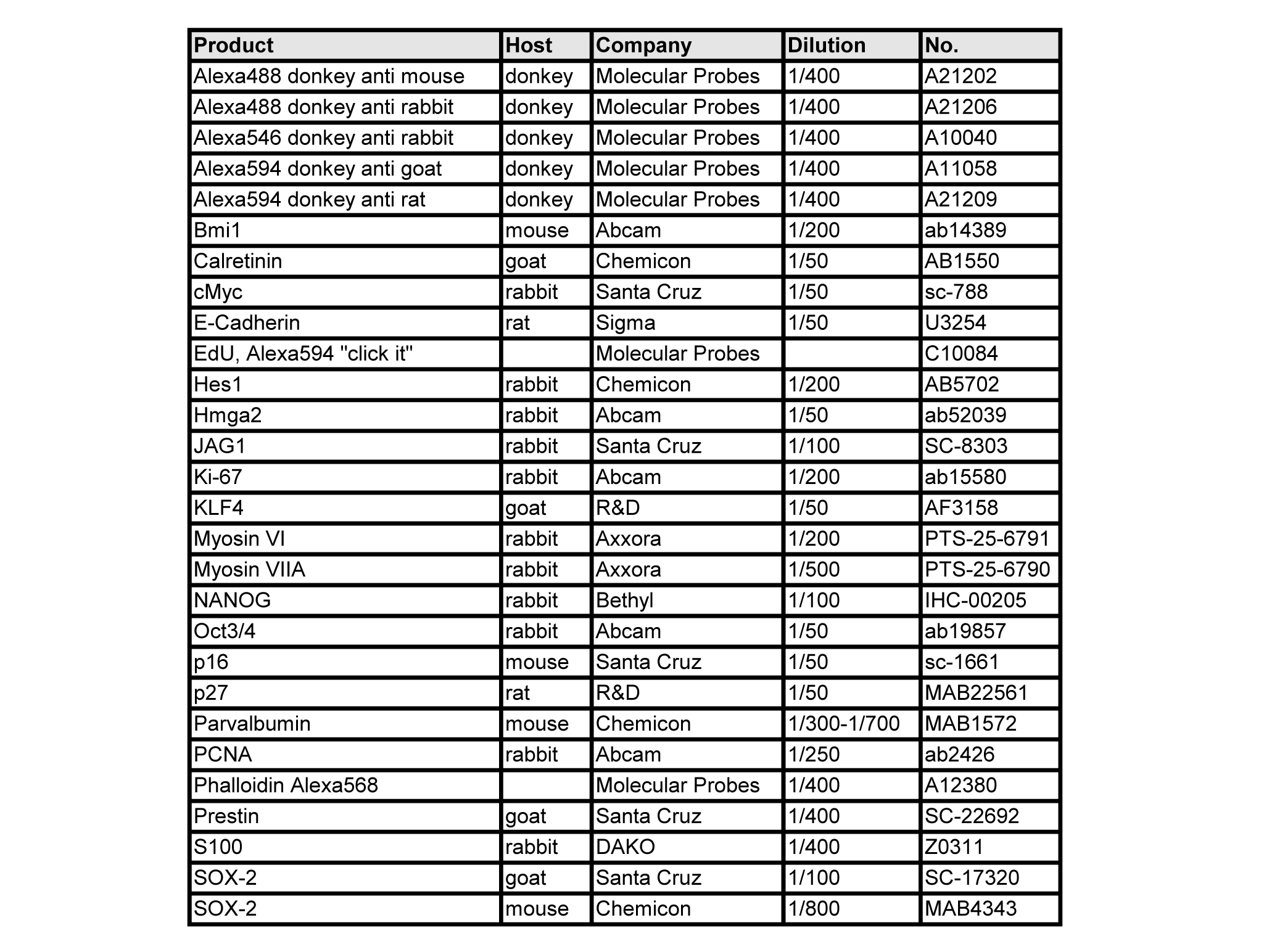

Supplement: Table S3 — Antibodies and fluorophores used in the study. (TIF) [file pone.0036066.s009.tif]
