# Supplementary material for: Stemness of the Organ of Corti Relates to the Epigenetic Status of Sox2 Enhancers
Source: PLoS One. 2012 May 3;7(5):e36066. doi: 10.1371/journal.pone.0036066 (PMC3343037; doi:10.1371/journal.pone.0036066)
Supplement: Supplementary Experimental Procedures S1 — (DOC) [file pone.0036066.s010.doc]

**SUPPLEMENTARY EXPERIMENTAL PROCEDURES S1**

**ESC cultures**

ESCs were obtained from Millipore (mES, No: SF-CMTI-2) (Millipore, Billerica, MA), and ESGRO COMPLETE™ (Millipore) was used according to the manufacturer’s guidelines. Cells were cultured in ESGRO Complete Clonal Grade Medium on gelatin-coated culture flasks (Millipore) in an incubator set to 7.5% CO_2_ in humidified air at 37°C. 1x10^6^ cells were initially plated on one T25 flask, and when mES cells reached ~ 60% confluence, a 1:5 split with Accutase (Millipore) was performed.

**Neurosphere cultures from postnatal forebrain**

Neurosphere cells from the postnatal forebrain were cultured as described previously[1,2]. The forebrains of C57BL/6 mice (P4) were dissected and enzymatically dissociated in 0.7 mg/mL of hyaluronic acid (Sigma-Aldrich, Munich, Germany) and 1.33 mg/mL of trypsin (Sigma-Aldrich) in HBSS (Invitrogen, Karlsruhe, Germany) with 2 mM glucose and buffered with HEPES (Invitrogen) at 37°C for 30 min. Addition of Earle’s Balanced Salt Solution (EBSS, Invitrogen) buffered with 20 mM of HEPES (Invitrogen) and 4% BSA (Sigma-Aldrich) stopped trypsinization. The cells were washed twice in 0.9 M sucrose (Sigma-Aldrich) in 0.56 M HBSS re-suspended in Dulbecco’s Modified Eagle Medium/F12 (DMEM/F12, Invitrogen) supplemented with B27 (Invitrogen), 2 mM glutamine (Sigma-Aldrich), 100 units/mL penicillin (Invitrogen) and 100 mg/mL streptomycin (Invitrogen) and buffered with 8 mM HEPES. 5,000 cells/mL were plated in uncoated tissue culture flasks (BD BiOCSCiences, Erembodegem, Belgium) and neurosphere formation was induced by adding 20 ng/mL of EGF (epidermal growth factor; Chemicon, Eagle Close, United Kingdom) and 10 ng/mL of FGF2 (fibroblast growth factor 2; Invitrogen. Neurosphere cells were characterized after 5 days *in vitro* culture.

**Otosphere cultures from postnatal OC**

Organ of Corti derived spheres from C57/BL6 mice were isolated as described previously[3]. 10 OCs were dissected per preparation and enzymaticly dissociated in 100µl of 0.125% trypsin in PBS at 37°C for 15 minutes. Addition of 50 µL containing 20 mg/mL soybean trypsin inhibitor and 2 mg/mL DNAse I solution (Worthington) in DMEM/ F12 media (Invitrogen) stopped the digest. After trituration cells were passed through a 70-mm cell strainer (BD Labware, Erembodegem, Belgium) and cultured in 10cm plastic Petri dishes (Greiner, Frickenhausen, Germany) containing 10ml of Dulbecco's Modified Eagle Medium (DMEM)/high glucose and F12 media (mixed 1:1) with N2 and B27 supplements (Invitrogen), bFGF (10 ng/mL), IGF-1 (50 ng/mL) and heparin sulfate (50 ng/mL) (growth factors obtained from R&D).

**Otosphere numbers and diameters**

Sphere numbers and diameters were analyzed using a CASY® TT cell counter and analyzer, equipped with a capillary 150 µm in diameter. The analyzed range was set from 25.2 to 60 µm. Results from 7-8 independent experiments were analyzed by Student’s t test using Jmp 6.0.2 (SAS, Cary, NC).

**Bisulfite conversion and sequencing**

Genomic DNA sodium bisulfite conversion was performed using an EZ-96 DNA Methylation Kit (Zymo Research, Orange Country, CA). The manufacturer’s protocol was followed, using 1 µg of genomic DNA and an alternative conversion protocol (a two-temperature DNA denaturation: 95ºC for 30 sec followed by 50ºC for 15 min; 20 cycles were performed, followed by storage at 4ºC). A Sequenom MassARRAY platform was used to perform a quantitative methylation analysis. This system utilizes matrix-assisted laser desorption ionization time-of-flight (MALDI-TOF) mass spectrometry with RNA base-specific cleavage (MassCLEAVE). A detectable pattern is then analyzed to determine methylation status. PCR primers were designed using EpiDESIGNER software (Sequenom, San Diego, CA). When feasible, amplicons were designed to cover CpG islands in the same region as the 5-UTR. For each reverse primer, an additional T7 promoter tag for *in vivo* transcription was added, as well as a 10-mer tag on the forward primer to adjust for melting temperature differences. MassCLEAVE biochemistry was performed as previously described[4]. Mass spectra were acquired using a MassARRAY Compact MALDI-TOF (Sequenom), and the spectra methylation ratios were generated by Epityper software v1.0.5.17 (Sequenom).

**Real-time PCR**

OCs from three developmental time points (E13.5, P4 and P21) and six different cell culture populations (ESC, NSC, OCSC, OCSC treated with EGF and differentiated OCSC [14 and 28 DIV]) were defined as biological groups, and each was investigated by three independent experimental replicates. Samples were dissected/isolated and immediately frozen in liquid nitrogen before lysis for RNA isolation. Total RNA isolation and DNase I treatment was performed using an RNAqueous®-Micro Kit (AM1931) (Ambion, Austin, TX), and complimentary DNA synthesis was performed with a High-Capacity cDNA Archive Kit (4387406) (Applied BioSystems, Darmstadt, Germany), both according to the manufacturer’s instructions. Transcript levels were determined using the fluorescence-based Quant-iT^TM^ assays with a Qubit^TM^ Quantitation Platform (Invitrogen). For each qPCR amplification reaction, the template was adjusted to 5 ng of total RNA in a volume of 20 µL. Measurements were performed in triplicate, and a no-template blank served as the negative control. A TaqMan® Gene Expression Master Mix (Applied BioSystems, 4369510) and the following oligonucleotides were designed by Taqman Assay-on-Demand (Taqman primer ID): Sox2 (Mm00488369_s1), Oct4 (Mm00658129_gH), Klf4 (Mm00516104_m1), cMyc (Mm00487803_m1), Nanog (Mm01617761_g1), Prox1 (Mm00435971_m1), p27Kip1 (Mm00438168_m1), Atoh1 (Mm00476035_s1), Myosin VIIa (Mm00485371_m1), Ubiquitin C (Mm00486943_m1), HPRT1 (Mm01298912_m1) and TbP (Mm01277045_m1). Assay-specific PCR efficiencies were calculated from the slope of standard curve experiments (Figure S2). C_T_ values were determined by the StepOnePlus^TM^ System using StepOne v2.1 software (Applied Biosystems), and PCR conditions were chosen according to the manufacturer’s manual. Transcription levels (Table S2) were normalized to the average of two housekeeping genes with the formula ΔΔ C_T_ using the qBasePlus software algorithm (Biogazelle, Zwijnaarde, Belgium). To choose the reference genes, three different housekeeping genes were analyzed (Ubiquitin C, HPRT1 and TbP), from which the two most stable reference genes were determined using the geNorm algorithm (Table S2) in qBasePlus software (Biogazelle).

**Statistical analysis**

Analysis of variance (ANOVA) and statistical comparisons were performed using qBasePlus software (Biogazelle) with the statistical significance set at 0.05 (Table S2). Finally, the data were calibrated to the biological reference group. Data are presented as means ± SDs. The heat map analysis of the transcriptional data was performed by using DataAssist 2.0 software (Applied Biosystems).

Criteria used to assign developmentally regulated genes to early, transition or differentiation groups

Developmentally regulated genes were assigned to early, transition or differentiation groups based on the following criteria. Early genes were expressed in the OC at E13.5 and significantly (p < 0.05) downregulated at P4 (Figure S2, Table S2B). Transition genes were transiently expressed at E13.5 and/or P4 and significantly (p < 0.05) downregulated at P21 (Figure S2, Table S2B). (3) Differentiation genes were expressed at the onset of differentiation at P4 and continued to be expressed to P21. Changes in expression from E13.5 to P4-P21 were statistically significant (Figure S2, Table S2B). According to the manufacturer’s manual, CT > 35 approached the sensitivity limits of the real-time detection system; thus, results with a CT > 35 were not included in the analysis. Generally, a gene was considered a low-copy gene when its expression level was within a CT range of 30 to 35. High-copy genes were those with CT values < 30.

**SUPPLEMENTARY REFERENCES**

1. Heinrich C, Blum R, Gascon S, Masserdotti G, Tripathi P, et al. (2010) Directing astroglia from the cerebral cortex into subtype specific functional neurons. PLoS biology 8: e1000373.

2. Johansson CB, Svensson M, Wallstedt L, Janson AM, Frisen J (1999) Neural stem cells in the adult human brain. Experimental cell research 253: 733-736.

3. Oshima K, Grimm CM, Corrales CE, Senn P, Martinez Monedero R, et al. (2007) Differential distribution of stem cells in the auditory and vestibular organs of the inner ear. Journal of the Association for Research in Otolaryngology : JARO 8: 18-31.

4. Ehrich M, Nelson MR, Stanssens P, Zabeau M, Liloglou T, et al. (2005) Quantitative high-throughput analysis of DNA methylation patterns by base-specific cleavage and mass spectrometry. Proceedings of the National Academy of Sciences of the United States of America 102: 15785-15790.
